# Development of an autonomous biosampler to capture *in situ* aquatic microbiomes

**DOI:** 10.1371/journal.pone.0216882

**Published:** 2019-05-15

**Authors:** Hugo Ribeiro, Alfredo Martins, Marco Gonçalves, Maurício Guedes, Maria Paola Tomasino, Nuno Dias, André Dias, Ana Paula Mucha, Maria F. Carvalho, C. Marisa R. Almeida, Sandra Ramos, José Miguel Almeida, Eduardo Silva, Catarina Magalhães

**Affiliations:** 1 CIIMAR–Interdisciplinary Center of Marine and Environmental Research, University of Porto, Av. General Norton de Matos s/n, Matosinhos, Portugal; 2 Institute of Biomedical Sciences Abel Salazar (ICBAS-UP), University of Porto, Porto, Portugal; 3 INESC TEC–INESC Technology and Science, Porto, Portugal; 4 ISEP–School of Engineering, Polytechnic Institute of Porto, Porto, Portugal; 5 Institute of Estuarine and Coastal Studies, University of Hull, Hull, United Kingdom; 6 FCUP–Faculty of Sciences of University of Porto, Porto, Portugal; Guangdong Technion Israel Institute of Technology, CHINA

## Abstract

The importance of planktonic microbial communities is well acknowledged, since they are fundamental for several natural processes of aquatic ecosystems. Microorganisms naturally control the flux of nutrients, and also degrade and recycle anthropogenic organic and inorganic contaminants. Nevertheless, climate change effects and/or the runoff of nutrients/pollutants can affect the equilibrium of natural microbial communities influencing the occurrence of microbial pathogens and/or microbial toxin producers, which can compromise ecosystem environmental status. Therefore, improved microbial plankton monitoring is essential to better understand how these communities respond to environmental shifts. The study of marine microbial communities typically involves highly cost and time-consuming sampling procedures, which can limit the frequency of sampling and data availability. In this context, we developed and validated an *in situ* autonomous biosampler (IS-ABS) able to collect/concentrate *in situ* planktonic communities of different size fractions (targeting prokaryotes and unicellular eukaryotes) for posterior genomic, metagenomic, and/or transcriptomic analysis at a home laboratory. The IS-ABS field prototype is a small size and compact system able to operate up to 150 m depth. Water is pumped by a micropump (TCS MG2000) through a hydraulic circuit that allows *in situ* filtration of environmental water in one or more Sterivex filters placed in a filter cartridge. The IS-ABS also includes an application to program sampling definitions, allowing pre-setting configuration of the sampling. The efficiency of the IS-ABS was tested against traditional laboratory filtration standardized protocols. Results showed a good performance in terms of DNA recovery, as well as prokaryotic (16S rDNA) and eukaryotic (18S rDNA) community diversity analysis, using either methodologies. The IS-ABS automates the process of collecting environmental DNA, and is suitable for integration in water observation systems, what will contribute to substantially increase biological surveillances. Also, the use of highly sensitive genomic approaches allows a further study of the diversity and functions of whole or specific microbial communities.

## 1. Introduction

Life in aquatic environments, including marine and freshwater ecosystems is dominated by a vast diversity and abundance of microorganisms. The whole marine microbial communities including phyto and zooplankton, bacteria, archaea, unicellular eukaryotes, protozoans and fungi are estimated to account for more than 90% of the total oceanic biomass. The activities of complex marine microbial communities are fundamental for the survival of all marine life [1,2]. Microorganisms can improve the water quality by naturally controlling the flux of nutrients, and also by degrading and recycling anthropogenic organic and inorganic contaminants [3–5]. Moreover, imbalances in plankton microbial communities, usually caused by environmental shifts can compromise water quality and all associated uses [6]. Hence, there is a great interest and need to study planktonic microbial communities on relevant temporal and spatial scales, to characterize their diversity and functional dynamics using the currently available highly sensitive genomic approaches.

The traditional method of microplankton sampling implies the collection of determined volumes of water at a pre-determined depth, usually using Niskin bottles in an individual fashion or in a rosette configuration at on-vessel crane [7]. These sampling methods involve also a manual water filtration step on board or at a home laboratory. This procedure increases costs mainly due to the rental and operation of the vessel, promotes deterioration of the sample [8] and increases the risk of potential contamination [9] derived from the storage time until the filtration step. In fact, it has been reported that gene expression profiles from samples collected and preserved *in situ* were significantly different from samples collected using Niskin bottles and preserved on deck [8].

To date, few autonomous biosampler systems have been developed, namely a prototype for sampling and preserving distinct biological class sizes that collects samples at low cost and is suitable to be adapted to a variety of vehicles [10]. However, this system is expensive to deploy, needs priori knowledge of the bio-life to be collected, requires high maintenance, and size limits its integration in smaller autonomous unmanned vehicles (AUV). A system for water collection through an AUV is also available [11], but this approach is limited to small volumes of water without the ability to concentrate water samples, limiting the use of those samples for highly sensitive analytic genomic approaches. Other systems were also developed for *in situ* and real time detection of specific genetic targets using automated sampling and molecular techniques to enumerate the abundance of specific species and functional groups [12–14]. However, these systems are extremely costly and limited to the identification of a particular protein, toxin and/or organism.

In this context, the aim of this study is to develop an *in situ* automatic bio-sampler system (IS-ABS) to study the plankton microbiome. IS-ABS was designed to collect and preserve *in situ* planktonic communities for further genomic, metagenomic, and/or transcriptomic analysis. We hypothesized that the microbiome collected with the IS-ABS will not differ from that collected with conventional manual sample methodologies [15], being suitable for highly sensitive analytical genomic approaches (genomic, metagenomic and transcriptomic) through massive sequencing analysis. For that, the reproducibility, environmental DNA (eDNA) recovery and diversity of prokaryotic (16S rDNA) and eukaryotic (18S rDNA) communities through massive sequencing analysis of samples collected by IS-ABS and manual standard filtering procedures were evaluated.

## 2. Material and methods

### 2.1 Conceptual approach of IS-ABS

The main objective of the IS-ABS system was to automate the steps and procedures traditionally performed in oceanographic campaigns, such as the Ocean Sampling Day (OSD) [15]. In particular, we aim to carry to the field, in an autonomous way, the manual sampling and laboratory filtration methods and techniques currently described in MicroB3 OSD Handbook [15]. This will reduce the logistical and operational costs of biological studies in the aquatic environments and will take advantage of current advanced technologies to improve both the quality of data gathering and its efficiency.

**Fig 1:**
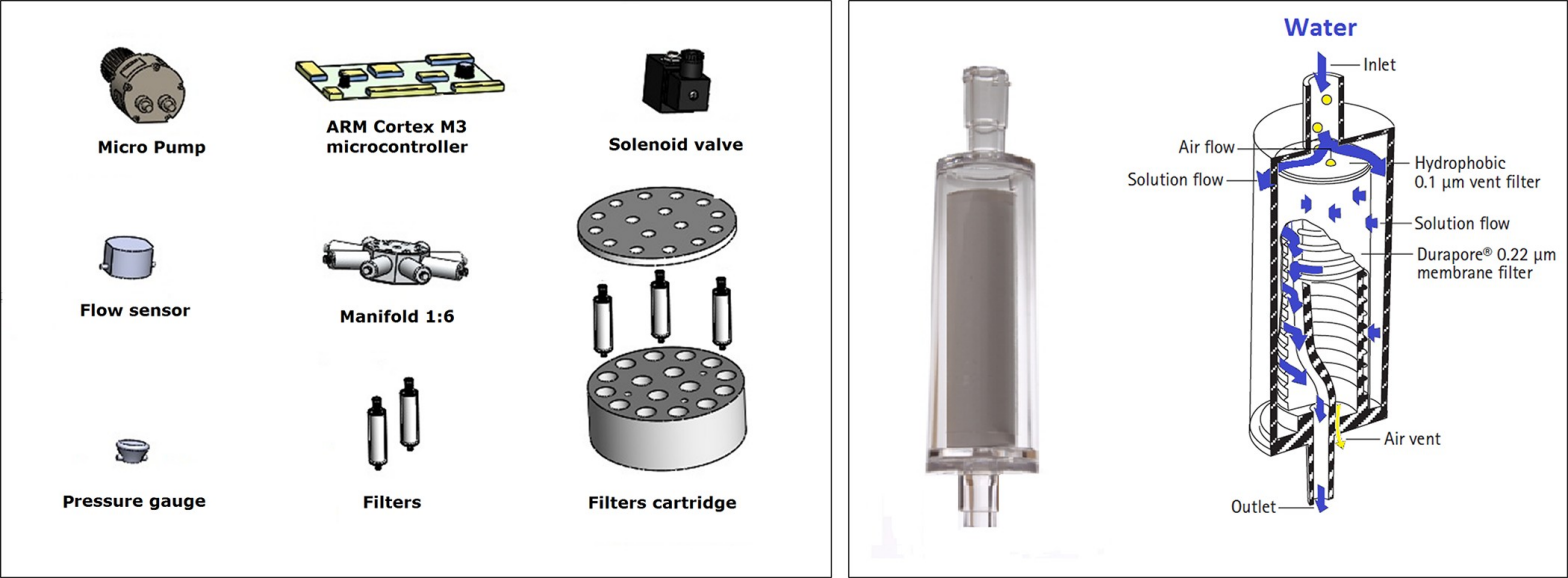
Electronic, micro-hydraulic and filters components. A) System components such as the pump, microcontroller, solenoid valve, flow sensor, manifold, pressure gauge, filters and filters cartridge used in the development of the IS-ABS. B) Sterivex-GP filter image (left) and Sterivex-GP filter diagram and water flow (right) during filtration procedure (adapted from User Guide [15]).

The IS-ABS consisted in a set of electronic and micro-hydraulic components and circuits for *in situ* water sampling and filtering, comprising several components, namely: a self-priming water pump (TCS MG2000), an ARM Cortex M4 microcontroller (STM32F411RE), a generic 100 A electronic speed controller (ESC) module, a flow sensor (Bio-Tech BT PCH-M-POM-LC 6), a Manifold 1:6 (NRESEARCH HP225T052), an analog pressure gauge (AVS-ROEMER E301), semi-rigid tubes for all wet circuits, push-in connections for all tubes, a set of filters and their cartridge (Fig 1A). The system was configured to use the same type of filters, the Sterivex-GP filter (Fig 1B), used in standard laboratory procedures [15]. This filter is a sterile device used to collect plankton organisms, particles, precipitates and undissolved powders larger than the chosen pore size, e.g. 0.22 μm [16]. The filter is non-toxic, self-venting, and capable of withstanding pressures up to 45 psi (≈3 bar). The Sterivex-GP filter has a reduced size and can be arranged in the IS-ABS in convenient multiple filter cartridge.

The system integrates full electronic control allowing a precise control and monitoring of the entire process. In addition, all the information on the performed sampling parameters and timestamp allows easy integration with data collected with other sensors. Embedded computer control is also relevant in order to integrate the system on autonomous systems such as AUVs (Autonomous Underwater Vehicles).

### 2.2 Architecture of the IS-ABS

**Fig 2:**
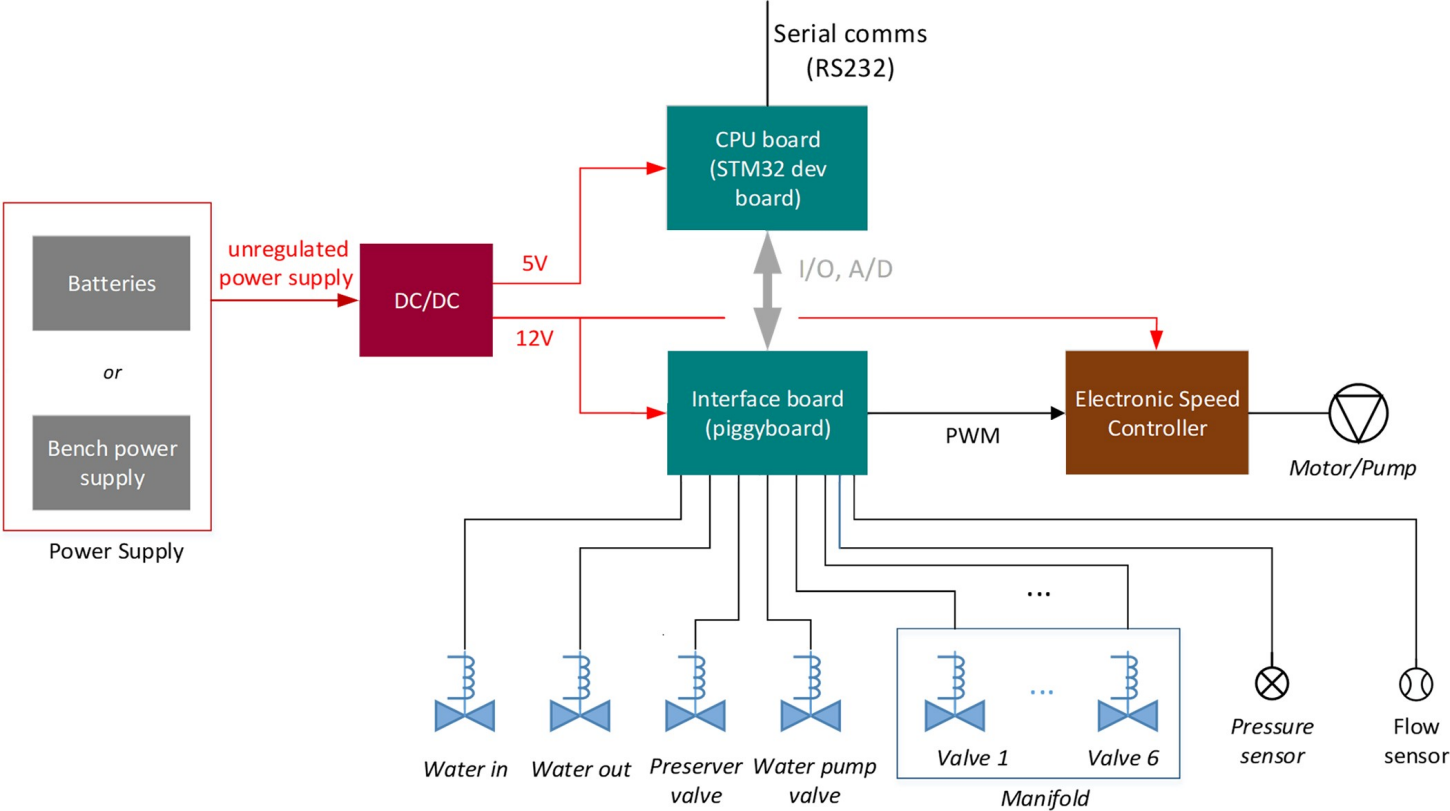
System control architecture. Main electronic blocks developed and how they connect to each other.

The IS-ABS control and programming was implemented in a two level hierarchical architecture (Fig 2). A low level microcontroller is responsible for the control of the micro-hydraulics circuit and related sensing. This system provides a set of functionalities that can be programmed/defined from a higher level control computer.

The water filtration system embedded control system was based on the STM32F411RE ARM Cortex M4 microcontroller running a Real Time Operating System (FreeRTOS). The microcontroller receives the high level mission definition through a RS232 communication line from a low power computer system. This computer system was based on an Odroid XU4 running Linux and has a set of databases which contains information of the tasks to be performed, as well as the status of the current filtering process and the logs of the previous filtering. This computer adjusts its clock via GPS when it is at surface and estimates the depth of the system using a pressure sensor. The microcontroller controls the opening and closing of the valves and the speed of the water pump.

Power supply can be provided externally (e.g. through an unregulated cabled DC source or by a lab bench power supply) or with a set of batteries. All the required regulated voltage lines for its components are produced in the system.

**Fig 3:**
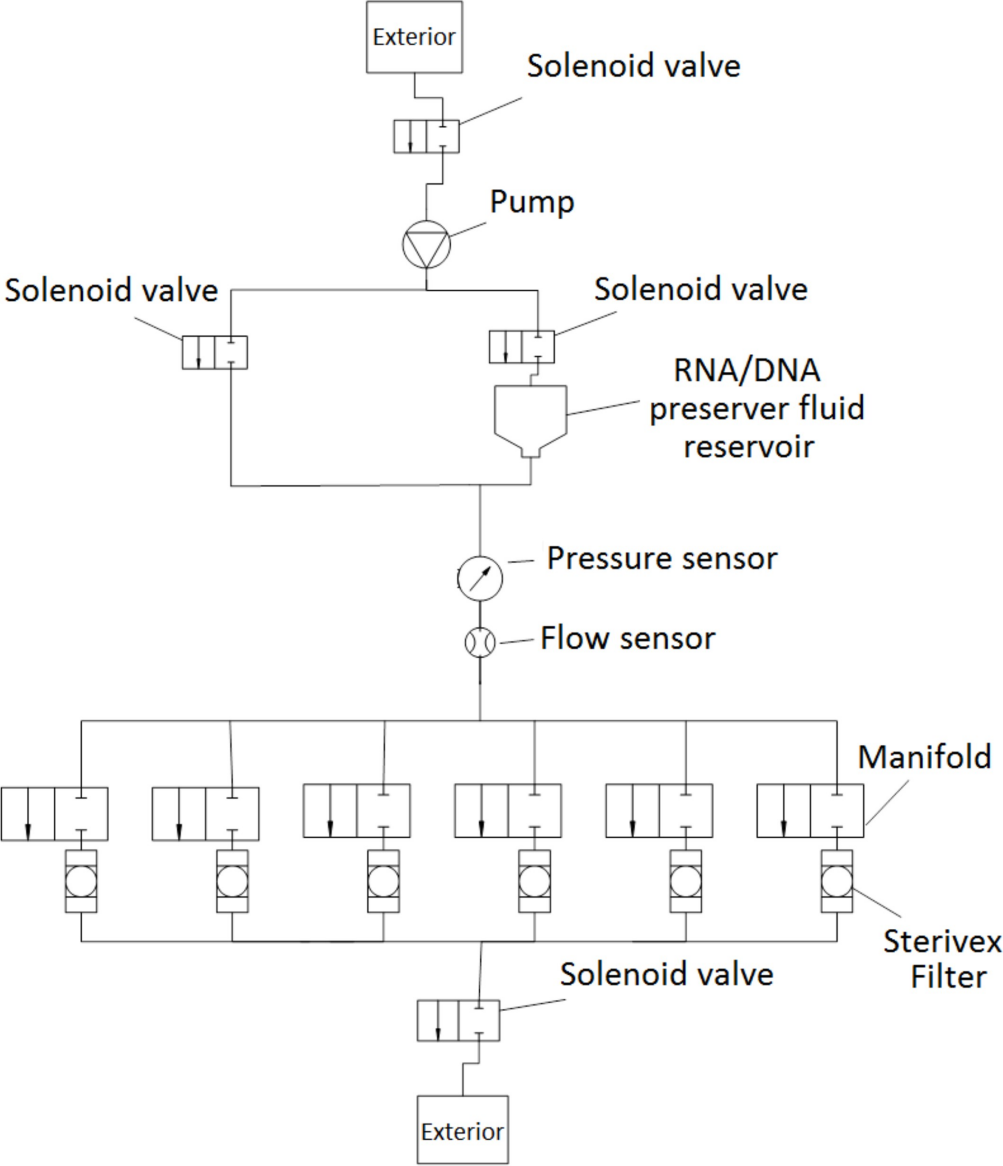
System hydraulic diagram. Water and RNAlater circuit with the solenoid valves that control which circuit is being used and the relative location of the pump and sensors.

The hydraulic circuit is represented in Fig 3 (only one 6 filter manifold is exemplified). The water is pumped from the environment to one or more (replicates) Sterivex filters with the pump controlled with an Electronic Speed Controller (ESC) via a Pulse-Width modulation signal (PWM), through the hydraulic circuit. These filters are selected by a set of valves arranged in the manifolds grouping six elements. Multiple manifolds can be used to select the desired number of filters. After water filtration, the pump can inject into the filter a preserving DNA/RNA solution from an onboard reservoir. Pressure and flow sensors allow controlling both filtration pressure and liquid flow to the filters (in both stages). An empty filter line (pass-through) is used to flush and clean the circuit.

**Fig 4:**
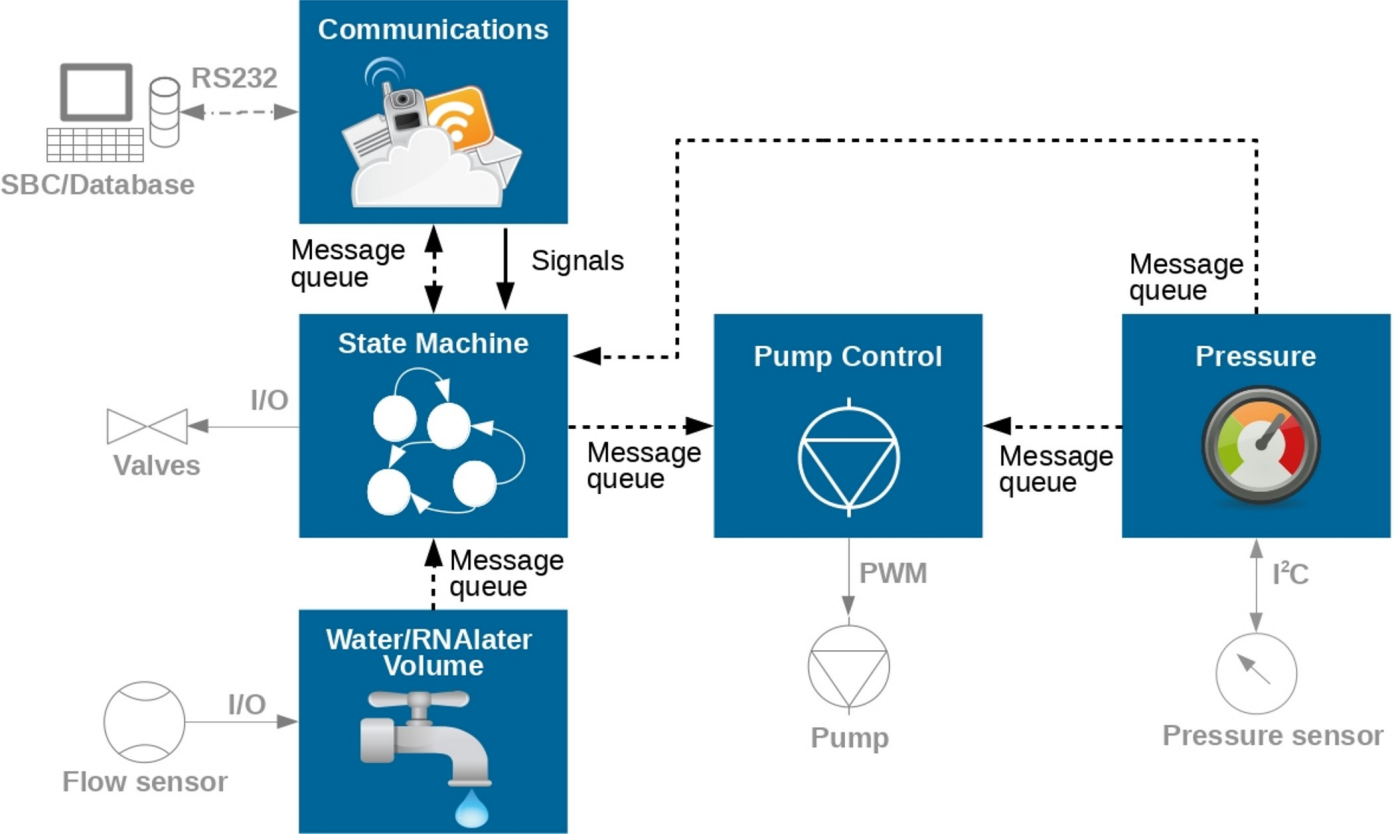
Embedded microcontroller software structure. Representation of the five tasks that are running in the microcontroller and which sensor or actuator is being connected to it. The information between them passed though a set of message queues and global event bits.

The embedded firmware was based on the FreeRTOS (Fig 4), a Real Time Operating System (RTOS) for the ARM Cortex M3, and starts by initializing all peripherals attached to the microcontroller. Peripherals include the pump, which has a PWM output, the valves that use an Input/Output (I/O), and the pressure sensor which has an analog output and is connected to the microcontroller 12-bit internal ADC and to the RS232 communication through the main board (Fig 4).

There are 5 tasks (or threads) running in the Real Time Operating System (communications, state machine, water/RNAlater volume, pump control and pressure). This implementation allowed for new feature integration since everything is contained in a separated task.

**Fig 5:**
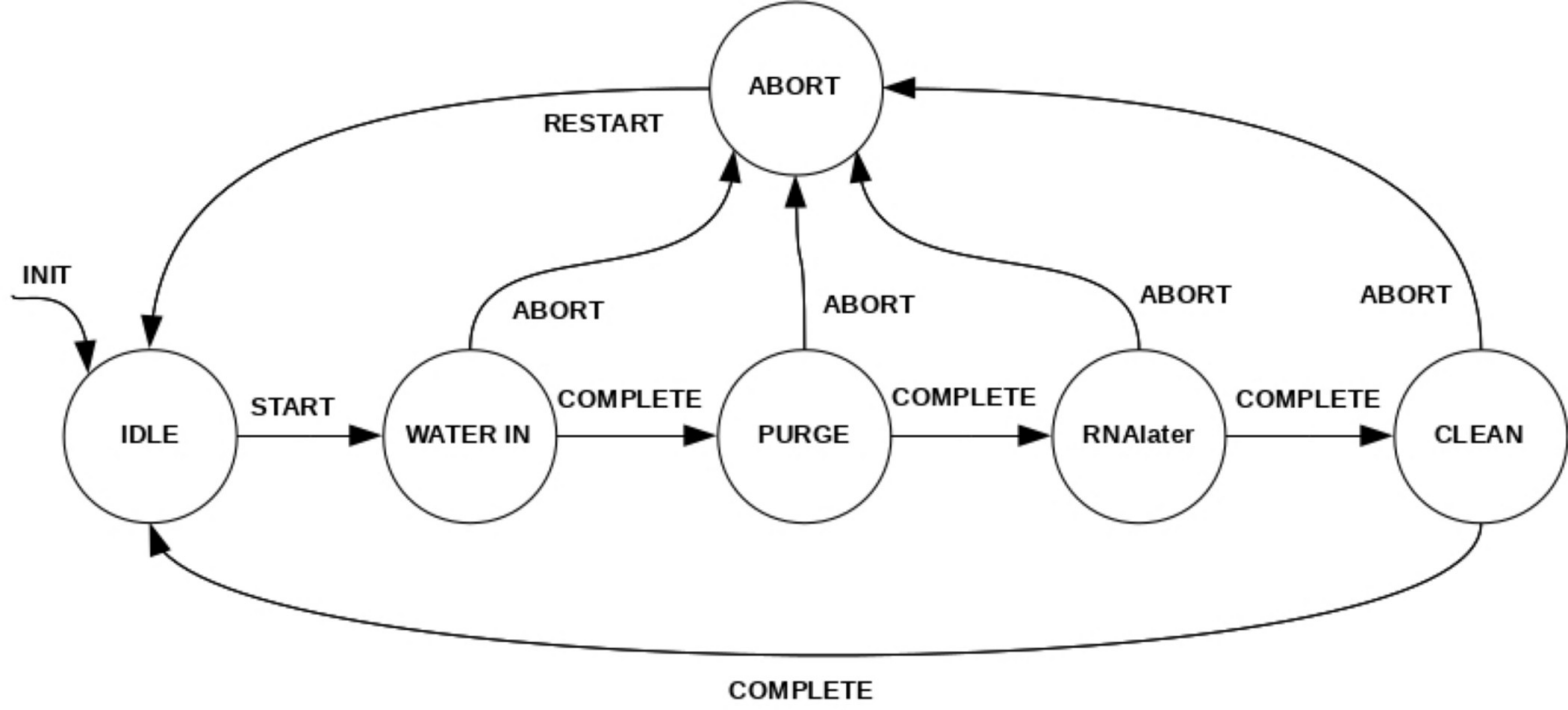
Embedded control software state machine. State machine implemented in the microcontroller firmware for global control of the system. The flags START COMPLETE and ABORT are set with information from the tasks that are processing the sensors signal.

The task *Communications* is responsible for reading the commands sent over RS232 by the main computer (SBC). These commands, after parsed, are passed into the correspondent task using the RTOS signals and/or message queues. The commands are mainly START or STOP actions for the filtration process and the configuration parameters. The *State Machine* task implements the state machine described in Fig 5 and is responsible for the global system coordination and control. This task blocks until a START command arrives and, during its execution, receives sensor data via message queues from other tasks that are used to change its current state. The data that comes from the task *Water/Preserved solution Volume* calculates the volume of water filtered and the amount of preserved solution injected into the Sterivex filter after filtration. The pump control is performed in another task, *Pump Control* that is solely responsible for controlling the pump according with the current system state (the pump is only activated in the WATER IN, PURGE, INJECT and CLEAN states). This pump controlling task receives inputs from the task *Pressure* that reads the pressure sensor and processes its signals to obtain the pressure applied by the pump to the Sterivex filters.

**Fig 6:**
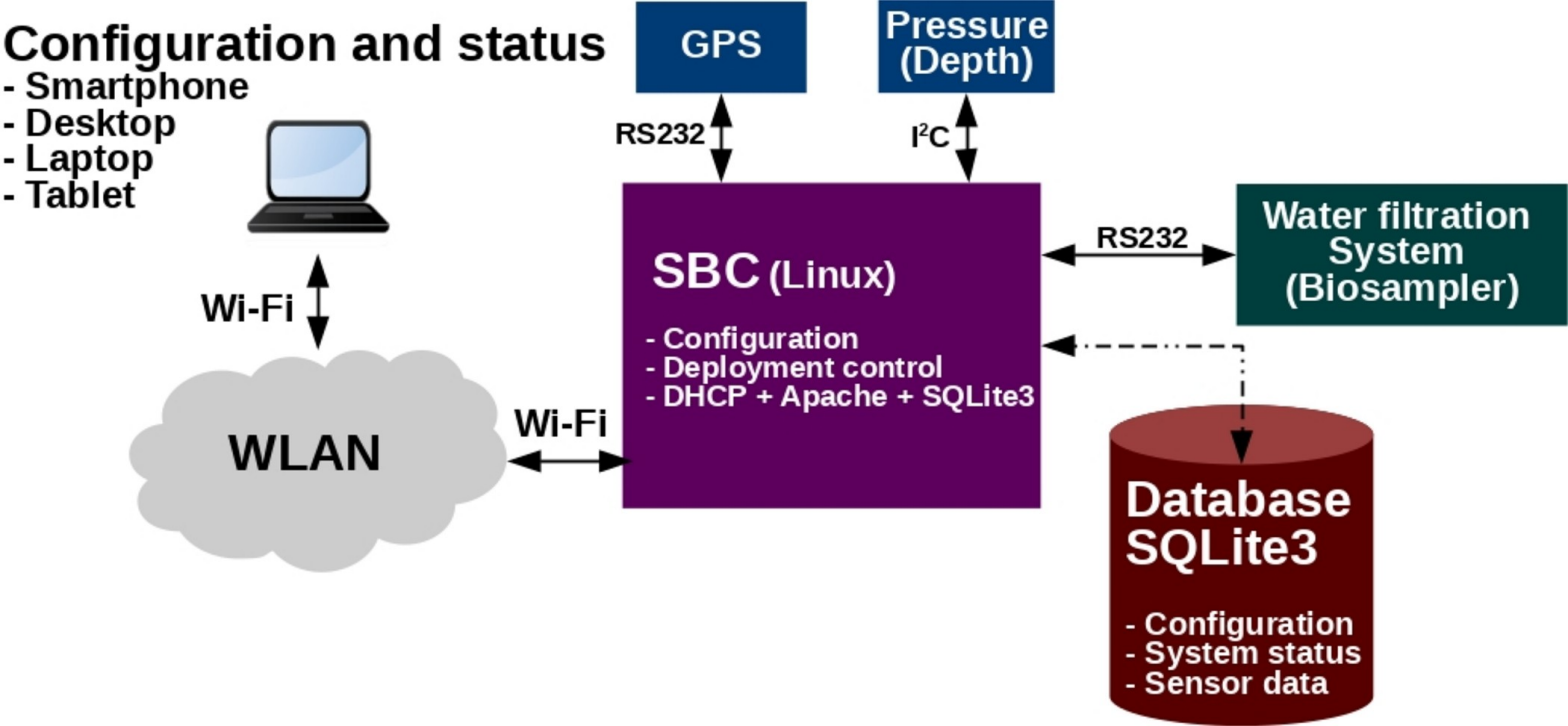
High level control and configuration. The user interface is based on a web page where the mission is configured and then saved on a local database (SSD disk). The mission in then passed to the embedded electronics through a RS-232 protocol.

An external environment pressure sensor allows estimating the depth and is available from the water filtration system electronics being its values obtained by the low power computer over I2C. The GPS is connected directly to the Single Board Computer (SBC) which synchronizes the clock using the Chrony service (Fig 6). The SBC allows a flexible development and future integration of other sensors that may require (e.g. saving a huge amount of data or having special communication protocols). Currently, the SBC provides a web interface based on PHP and SQLite3 using a Wi-Fi antenna that allows to input the parameters to the filtering operation as well to monitor the current status of the biosampler (when it is at the surface). The mission can be configured by using any device with Wi-Fi and a web browser such as Smartphone, Desktop, Laptop or Tablet, allowing simple and fast setup of the filtration operation (Fig 6).

A filtration mission can be configured by the user by pre-setting a set of input parameters controlling the filtration process. These parameters include: (i) volume of water to be filtered; (ii) maximum pumping pressure; (iii) water column depth at which the filtration should start; (iv) number of simultaneous samples to be collected by filtration; (v) time of the day to start the filtration mission (e.g. 17pm). The mission is configured by entering the number of Sterivex filters available in the cartridge, the initial time of the sampling, the delay between collection of samples and how many replicates should be taken. This is done with a device with an internet browser that connects via Wi-Fi to the SBC. The SBC has a HTML server (Apache) with a configuration web page (S1 Fig) and saves every configuration provided by the user as well as the sensor data in a SQLite3 database.

The configuration is then encapsulated by a service written for this purpose that runs in the operating system providing a simple interface for the user and also returning a simple feedback resume of the operation to be executed. The operation setup is then passed to the microcontroller via RS232 protocol.

#### 2.2.1 Tests of filtration volumes *vs* time performance

The performance of the IS-ABS in terms of filtration volumes and time was assessed by monitoring the filtration time of 2 L of water at three distinct constant working pressures (0.8, 1.3 and 1.8 bar). Filtration time was measured at each 100 ml of water filtered until reaching a total filtration volume of 2 L.

### 2.3 Validation for microbiome analysis

The IS-ABS prototype was validated by performing parallel filtration with the IS-ABS and with a conventional OSD protocol [15], and by comparing the results in terms of plankton microbial community structure and eDNA recovered.

Surface seawater samples were collected in November 2016 at approximately 25 km offshore NW Portuguese coast (41.41 N; 09.18 W), on behalf of the National Biological Sampling Program of the Portuguese Institute for Sea and Atmosphere (IPMA). Samples were collected in washed plastic buckets, stored into two 20 L carboys (prewashed with MilliQ) and transported to the laboratory at CIIMAR for analysis following the procedures described in OSD guidelines [15].

#### 2.3.1 Filtration procedures

The OSD filtration apparatus (S2 Fig) consisted of a diaphragm vacuum pump (KNF N145 AN.18) linked to a water waste collection bottle, which receives filtered water from 50mL sterile syringes connected to a 0.22 μm Sterivex filter [15], and to a PowerVac Manifold (product 11991, MO-BIO Laboratories, Inc.). The vacuum pump has an ultimate vacuum of 100 mbar (abs), which creates a differential pressure of approximately 1 bar.

The IS-ABS filtration procedure used a peristaltic self-priming water pump (MG2000) and a 0.22 μm Sterivex cartridge as described in Section 2.1.

A total volume of 3 L of coastal seawater was filtered through each Sterivex filter unit. Filters were stored at -80°C until DNA extraction following the OSD guidelines [15].

**Fig 7:**
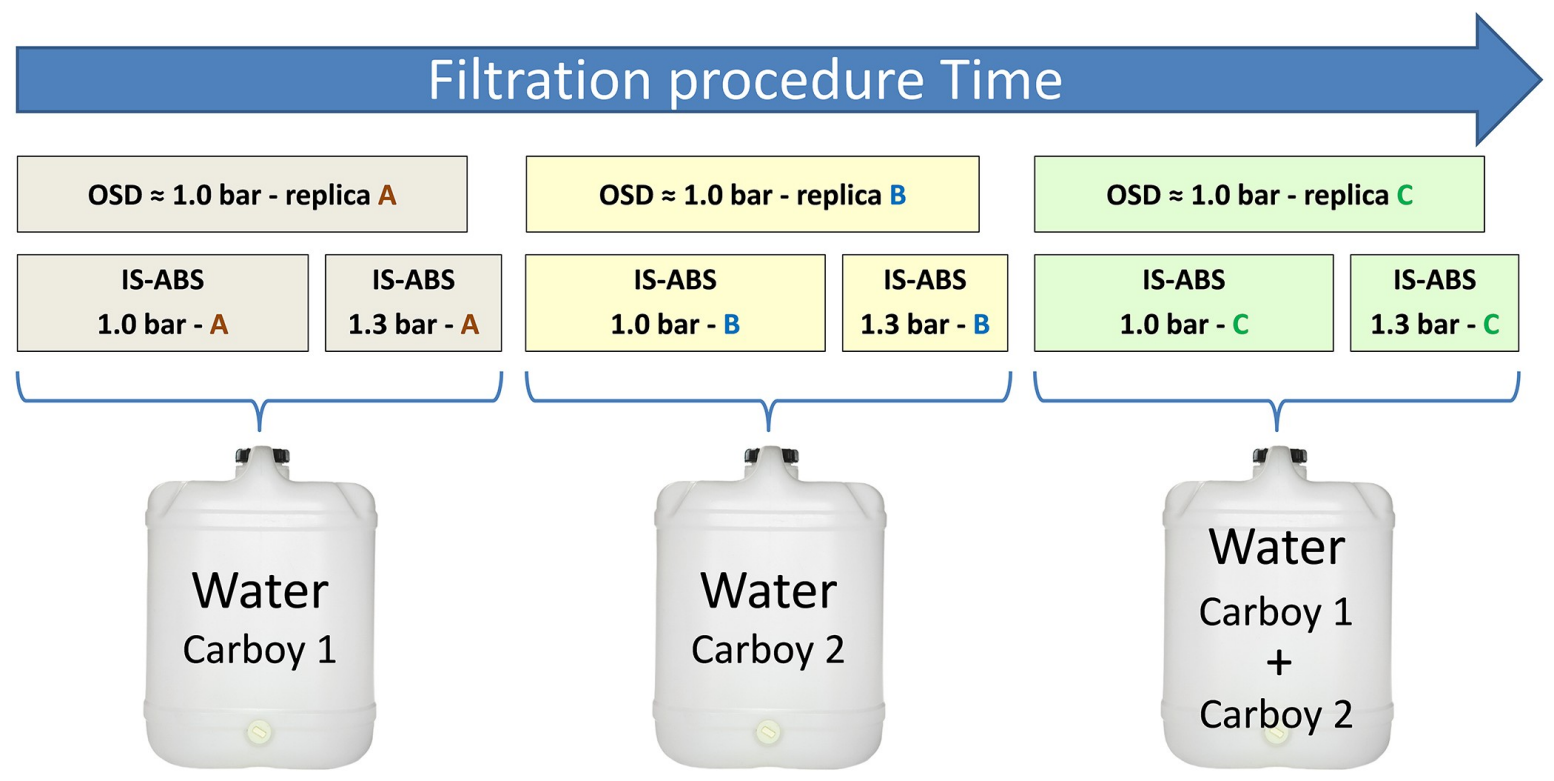
Filtration procedure design. The filtration of the different replicates started simultaneously in the OSD procedure and in the IS-ABS in the different carboys.

The comparison of OSD and IS-ABS filtration was carried out in triplicate (A, B, C) and at a similar filtration pressure (≈ 1.0 bar). For the IS-ABS, an additional filtration pressure (1.3 bar) was also included (Fig 7).

To avoid potential differences between the two filtration procedures due to filtration time lapse and/or differences caused by seawater storage in different carboys, replicate filtrations started simultaneously in both procedures (Fig 7). In addition, carboys were manually shaken immediately before each filtration to guaranty homogeneity of the sample.

#### 2.3.2 Microbiome analysis

DNA was extracted from each Sterivex filter using the PowerWater DNA Isolation Kit protocol (MO BIO Laboratories, Inc., Portugal) following the manufacturer’s instructions. Concentration and quality of DNA were measured by fluorometry using the Qubit fluorometric quantitation kit (Qubit dsDNA High Sensibility Assay Kit, Thermo Fisher Scientific). Environmental DNA obtained after extraction was used for 16S rDNA and 18S rDNA metabarcoding analysis targeting prokaryotes and eukaryotes, respectively. Hypervariable V4-V5 region (≈412 bp) of 16S rDNA was amplified using the universal primer pairs 515YF/Y906R-jed [17]. For eukaryotes V4 region (≈434 bp) of 18S rDNA was amplified using TAReuk454FWD1 / TAReukREV3_modified primers set [18]. Paired-end sequencing was performed on an Illumina MiSeq platform at LGC Genomics, Berlin, Germany, protocol detailed description is given in Ribeiro et al. [19].

Illumina paired-end reads were preprocessed independently (for each SSU rDNA library, i.e., 16S and 18S rDNA datasets) using mothur v.1.38.1 [20] following the MiSeq Standard Operating Procedure [21]. Primer sequences were removed, no ambiguous bases were allowed, the maximum homopolymer size was 8 bp. The remaining sequences were dereplicated and screened for chimeras using UCHIME in de novo mode [22]. Taxonomic assignment of the unique reads was performed independently for each SSU using a naïve Bayesian Classifier [23] against SILVA database (v. 1.2.8) for 16S SSU and the PR2 database for 18S SSU [24]. Nomenclature and terms of ranks of the PR2 database follow the classification of eukaryotes proposed by Adl et al. [25].

Afterward, VSEARCH de novo clustering algorithm was used to clusterize the sequences into OTUs (Operational Taxonomic Units) using 0.03 cutoff value for both 16S and 18S rDNA amplicon datasets [26] to build the 16S and 18S rDNA amplicon-based OTU tables. Global singletons and OTUs affiliating to metazoans were removed. OTU tables (one for each SSU rDNA library) produced through mothur pipeline were converted into biom format and imported to QIIME (QIIME 1.9.1; [27]) to perform the downstream analysis. The number of sequence reads per sample was rarefied by random sampling to the lowest read number sample, which was 23574 and 7654 respectively for 16S and 18S libraries to explore alpha (within samples) and beta diversity (between samples). Rarefaction curves of observed OTUs and *α*-diversity estimators (Chao1, Shannon–Wiener, Simpson-evenness) were obtained in QIIME [27].

Sequence clustering threshold at 97% was chosen in our workflow to cluster similar sequences into Operational Taxonomic Units (OTUs) at species level. OTUs affiliated to Metazoa were removed from the dataset because their presence in the Sterivex size fraction (<0.22 um) could be due to minute life cycle stages but also to the breakdown of animals during filtration [28].

Raw Illumina fastq files, concerning the SSU rDNA amplicon data used in this study, have been deposited to European Nucleotide Archive under the project accession number PRJEB27645.

#### 2.3.3 Data analyses

A comparative evaluation of microbial community structure detected by OSD and IS-ABS was performed focusing on both total community and on the 'rare biosphere' (i.e. the pool of low-abundance taxa found in the dataset). The cutoff for characterizing it as well as the methods is arbitrary [29]. In the present study, a relaxed definition for the rare biosphere has been adopted, by selecting from the total prokaryotic and eukaryotic OTUs tables respectively, a pool of the low-abundance species below the threshold of 1% of the total community [30,31].

Heatmaps and Spearman correlations were generated using Hmisc, corrplot and ggplot2 R packages [32–35] implemented in the ORCA platform [36].

Beta diversity of Prokaryotic and Eukaryotic communities were calculated using OTUs relative percentage values with PRIMER software (version 6.1.11) [37]. The community structure was investigated by cluster analysis (complete linkage method) using a Bray-Curtis similarity matrix and Simprof test to investigate significant (*P* < 0.05) differences between clusters.

Differences on diversity indices and relative abundance among the two filtration systems were analyzed by a 1-way ANOVA followed by multiple Tukey comparison test. Statistical analysis was performed with the software STATISTICA, version 7, StatSoft, Inc. (2004).

## 3. Results

### 3.1 IS-ABS mechanical integration and functioning

**Fig 8:**
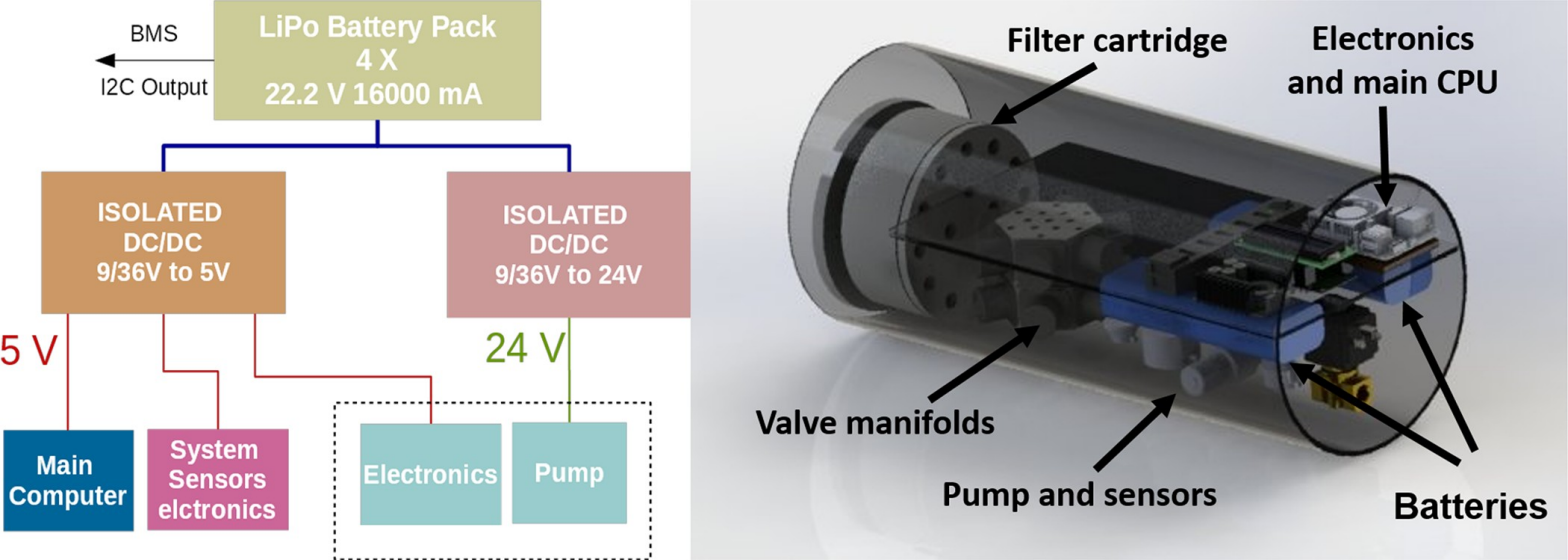
**Field autonomous biosampler prototype components (left) and CAD model (right).** The components are all mounted inside a cylinder in vacuum.

The IS-ABS prototype includes the hydraulics components (Fig 1A and Fig 3) such as the water pump, microcontroller, ESC module, flow sensor, Manifold 1:6, analog pressure gauge, semi-rigid tubes for all wet circuits, push-in connections for all tubes, and a set of filters and their cartridge, embedded controller electronics (Section 2.2), the main low power computer and a set of LiPo batteries (Fig 8).

**Fig 9:**
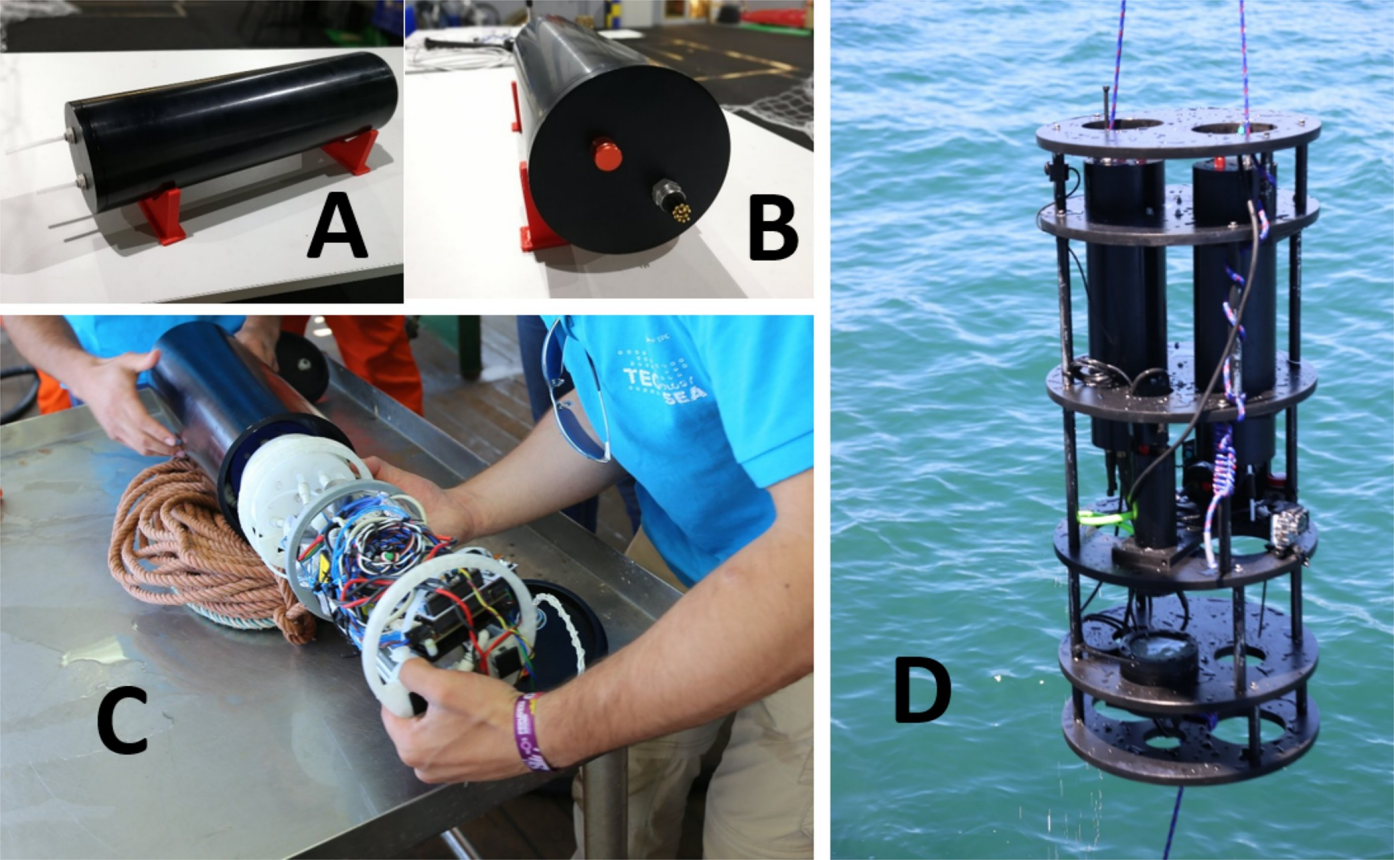
*In situ* autonomous biosampler (IS-ABS) prototype. Water inlet/outlet (A); external connector interface (B); opened in the field (C); integrated in a multi-sensor system.

All the components of IS-ABS were housed in a 150 mm diameter and 500 mm length aluminum pressure housing allowing for operation up to 150 m depth (Fig 9). For the hydraulic circuit, a set of flexible plastic tubes and fast connectors allowed for ease of maintenance and corrosion resistance. The standalone IS-ABS (Fig 9A, 9B and 9C) has an external underwater connector (Fig 9B) allowing for integration with other systems (Fig 9D), such as the MarinEye multiple sensor system [38].

The components of the hydraulic circuit, flexible plastic tubes and fast connectors, are transparent and can be placed under UV light for sterilization and elimination of eventual DNA from exogenous microorganisms [9]. Before the filtration mission these hydraulic circuit components can be easily set in the IS-ABS. The IS-ABS system operates as follows: first *in situ* water from the intended location, is pumped with a micropump (TCS MG2000) through the hydraulic circuit, and flushed throughout the system to clean eventual residues in the piping and valves. Then, the filtration process starts, and water is filtered *in situ* in one (controlled through the manifold system) or more (replicates) Sterivex filters placed in a filter cartridge.

The filtration process is controlled by the embedded control system according to the predefined parameters. Either the volume of water to be filtrated, the duration of the filtration process or even the detection of filter blocking can be used to end the process. Once the filtration ends, a DNA/RNA preserving solution is pumped into the filter to preserve the sample for posterior retrieval [39]. Depending of the sampling and mission requirements, the system can be expanded by adding groups of manifolds and filter cartridges to the prototype.

**Fig 10:**
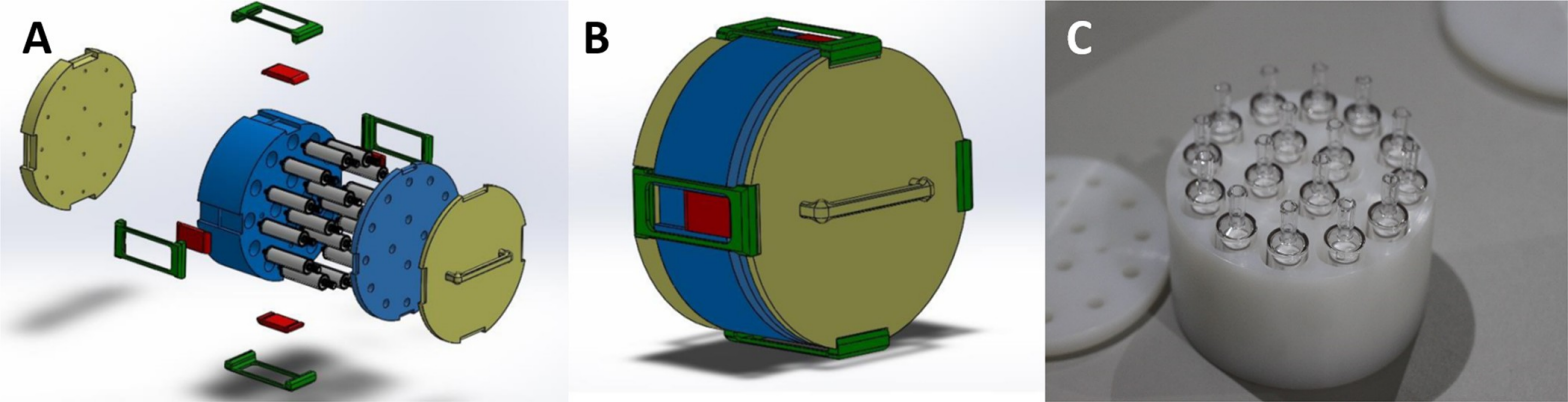
Filter cartridge box. Design of the filter cartridge box open (A) and closed (B); and Sterivex filter cartridge image (C).

The IS-ABS integrates a novel filters’ cartridge box, made by a set of pieces that couple together (Fig 10A and 10B) and specially designed to easily storage the cartridges with Sterivex filters. Thus, the cartridge can conveniently be taken out of the IS-ABS and sorted at the end of the filtration mission until eDNA extraction. This box, made in high-density polyethylene (HDPE 1000), houses a set of 16 filters within the cartridge (Fig 10) that can be removed individually or jointly, depending on the user’s option.

The power source of IS-ABS prototype is based on a pack of 4 LiPo batteries with 22.2 V and 16000 mA with low weight and high density. These batteries are connected to two isolated wide input and low noise output DC/DC converters with 5V and 24 V outputs respectively. From this point every subsystem receives the necessary voltage input. For the electronic systems that need other voltages, such as 3.3 V, the voltages are provided in the printed circuit board by low dropout voltage regulators. The batteries are not mandatory because the IS-ABS can be integrated in other systems (for instance in a Remote Operated Vehicle or an Autonomous Underwater Vehicle) that can provide the necessary power.

#### 3.1.1 Filtration flow performance

The initial assessment of the IS-ABS filtration performance showed that increasing the pump speed and concomitantly the pressure (from 0.8 to 1.3 and 1.8 bar) induced a higher average filtration flow and significantly lowered the filtration time considering the same volume (2 L) of water (S1 Table). Moreover, considering each filtration pressure tested, a significant (ANOVA, *P* < 0.05) decrease on the average flow with the increase of water volume filtrated or filtration time was recorded (S1 Table). Comparing with the manual procedure (35.8 ± 0.3 min), the IS-ABS system substantially decreased the filtration time (24 ± 1 min) when equal volume of water (2 L) was filtered (S1 Table).

### 3.2 IS-ABS validation for microbiome analysis

IS-ABS validation for plankton microbiome analysis was performed by evaluating the eDNA recovered and by examining microbial community diversity using high-throughput sequencing technologies [8].

#### 3.2.1 Performance on the DNA recovered

Results on the eDNA recovered after filtering 3 L of water at the same pressure (1 bar), using the standard OSD manual procedure and the autonomous IS-ABS system, showed a similar (*P* ≥ 0.05) eDNA recovered between these two methods (Table 1). The IS-ABS had the advantage of filtering samples in a shorter time (Table 1) due to its higher flow rates relatively to the manual OSD procedure.

**Table 1:** Filtration time, volume, average flow and environmental DNA recovered. In the tests performed with the Ocean Sampling Day (OSD) standard procedure and the autonomous biosampler (IS-ABS) (mean ± standard deviation, n = 3). For IS-ABS, two filtration pressures were selected. Different superscript letters indicate significant (ANOVA, *P* < 0.05) differences among the three filtration procedures for each parameter.

		OSD	IS-ABS	
**Pressure** (bar)		≈1	1	1.3
**Time of Filtration** (minutes)		128^a^ ± 16	61^b^ ± 4	56^b^ ± 5
**Mean Flux** (mL/min)		24^a^ ± 3	50^b^ ± 3	54^b^ ± 5
**eDNA recovered** (μg/mL)		7^a^ ± 5	7^a^ ± 2	10^a^ ± 8
**Volume per replicate** (L)		3	3	3

Comparing the two pressures tested with IS-ABS, no significant differences (ANOVA, *P* ≥ 0.05) were also observed between the amounts of DNA recovered, although at the higher pressure tested an increase of the variation (standard deviation) in eDNA concentrations was observed (Table 1).

#### 3.2.2 Performance on 16S and 18S rDNA sequences and OTUs recovered

DNA samples obtained from the different filtration tests (Section 3.2.1) were analyzed through metabarcoding to investigate prokaryotic (16S rDNA) and unicellular eukaryotic communities (18S rDNA). This procedure aimed to identify potential differences between manual and autonomous filtrations (OSD and IS-ABS) regarding the plankton microbial community structure. Moreover, a comparison between microbial structure and diversity of samples filtered by the IS-ABS at 1 bar and 1.3 bar was also performed.

A total of 540677 and 266117 raw read pair sequences were obtained as sum of both filtration procedures, respectively for 16S rDNA and 18S rDNA. Sorting procedure performed by mothur pipeline produced a total curated dataset of 462956 (16S) and 227045 (18S) unique sequences. Clustering the reads at 97% of similarity for both prokaryotes and eukaryotes produced 385029 and 149725 OTUs (S2 Table).

An overview of the sequences recovered (S2 Table), raw, filtered and then clustered, reveals no significant differences regardless the selected filtration system (OSD and IS-ABS prototype).

#### 3.2.3 Microbiome diversity

The reproducibility of the manual and autonomous (IS-ABS) filtration procedures on microbiome diversity was evaluated by comparing several diversity indices, including the number of observed OTUs, Chao1, Shannon, Berger Parker dominance, Simpson’s evenness, and also the Good coverage (Table 2). General trends in diversity indices calculated showed no significant (ANOVA, *P* > 0.05) differences regardless the filtration procedure tested (Table 2).

**Table 2:** Diversity indices for 16S and 18S rDNA. Calculated in the samples recovered using either the Ocean Sampling Day filtration standard procedure and the autonomous biosampler (IS-ABS) (mean ± standard deviation, n = 3). For IS-ABS two filtration pressures (1 and 1.3 bars) were used. Different superscript letters indicate significant (ANOVA, *P* < 0.05) differences among the three filtration procedures for each diversity index.

	Diversity indices	OSD	IS-ABS	
		≈1bar	1bar	1.3 bar
**16S rDNA**				
	Observed OTUs	2523^a^ ± 417	2390^a^ ± 228	2650^a^ ± 462
	Chao1	6370^a^ ± 2428	5589^a^ ± 253	7072^a^ ± 3096
	Shannon index	7.5^a^ ± 0.4	7.4^a^ ± 0.5	7.4^a^ ± 0.1
	Berger Parker	0.13^a^ ± 0.05	0.11^a^ ± 0.02	0.13^a^ ± 0.03
	Simpson’s evenness	0.014^a^ ± 0.008	0.015^a^ ± 0.004	0.012^a^ ± 0.004
	Good coverage	0.94^a^ ± 0.02	0.94^a^ ± 0.01	0.93^a^ ± 0.02
**18S rDNA**				
	Observed OTUs	583^a^ ± 220	648^a^ ± 60	625^a^ ± 77
	Chao1	773^a^ ± 352	912^a^ ± 78	831^a^ ± 189
	Shannon index	7.0^a^ ± 0.2	6.8^a^ ± 0.4	6.7^a^ ± 0.4
	Berger Parker	0.07^a^ ± 0.01	0.10^b^ ± 0.02	0.13^b^ ± 0.05
	Simpson’s evenness	0.09^a^ ± 0.04	0.06^a^ ± 0.01	0.06^a^ ± 0.04
	Good coverage	0.98^a^ ± 0.02	0.969^a^ ± 0.003	0.97^a^ ± 0.01

The convergence observed in the rarefaction curves reflected that almost all OTUs accumulated at a roughly constant rate as the number of reads increased (S3 Fig) and no different rarefaction curves were found among the different filtration procedures.

#### 3.2.4 Performance at high community taxonomy level

The analysis of prokaryotic and eukaryotic community structure (Part A and B in S4 Fig, respectively) at the phylum level did not show major differences regardless the different filtrations procedures (OSD protocol and IS-ABS prototype) at a similar working pressure. In fact, the occurrence of the dominant Archaea, Bacteria and unicellular Eukaryotes phyla recovered among samples, using either the OSD or the IS-ABS filtration procedures showed similar (ANOVA, *P* ≥ 0.05) relative percentage of OTUs (Table 3 for Archaea and Bacteria, Table 4 for Eukaryotes).

**Table 3:** Relative percentage (>1%) of 16S OTUs (Bacteria and Archaea) taxonomic composition at phylum level. Detected in the tests performed with the Ocean Sampling Day (OSD) standard procedure and with the autonomous biosampler (IS-ABS) (mean ± standard deviation, n = 3). For IS-ABS two filtration pressures were selected (1 and 1.3 bar). Different superscript letters indicate significant (ANOVA, *P* < 0.05) differences among the three filtration procedures for each phylum.

		OSD	IS-ABS	
		≈1bar	1bar	1.3 bar
**Relative percentage of main Bacteria Phyla**				
	*Alphaproteobacteria*	34^a^ ± 4	31^a^ ± 2	32^a^ ± 4
	*Flavobacteriia*	29^a^ ± 2	32^b^ ± 1	30^ab^ ± 2
	*Gammaproteobacteria*	14^a^ ± 2	14^a^ ± 2	13^a^ ± 1
	*Cyanobacteria*	2.3^a^ ± 0.4	2.7^ab^ ± 0.5	2.9^b^ ± 0.2
	*Planctomycetacia*	2^a^ ± 1	2.6^a^ ± 0.4	2.7^a^ ± 0.7
	*Acidimicrobiia*	2.5^a^ ± 0.5	2.0^a^ ± 0.5	2.5^a^ ± 0.4
	*Sphingobacteriia*	2.4^a^ ± 0.4	2.5^a^ ± 1	2.0^a^ ± 0.1
	*Verrucomicrobiae*	1.9^a^ ± 0.2	1.7^a^ ± 0.4	1.9^a^ ± 0.5
	*Deltaproteobacteria*	1.5^a^ ± 0.1	1.3^ab^ ± 0.2	1.2^b^ ± 0.2
	*Betaproteobacteria*	0.6^a^ ± 0.4	2.4^a^ ± 3.2	1.9^a^ ± 2.3
**Relative percentage of main Achaea Phyla**				
	*Thaumarchaeota*	0.2^a^ ± 0.1	0.11^a^ ± 0.03	0.2^a^ ± 0.1
	*Woesearchaeota*	0.06^a^ ± 0.04	0.06^a^ ± 0.02	0.1^a^ ± 0,1
	*Euryarchaeota*	0.07^a^ ± 0.01	0.05^a^ ± 0.02	0.10^a^ ± 0.04
	*Diapherotrites*	0.004^a^ ± 0.002	0.005^a^ ± 0.006	0.002^a^ ± 0.002
	*Bathyarchaeota*	0.003^a^ ± 0.003	0.004^a^ ± 0.006	0.002^a^ ± 0.004
	*Archaea* unclassified	0.004^a^ ± 0.001	0.003^a^ ± 0.002	0.003^a^ ± 0.003
	*Lokiarchaeota*	0.001^a^ ± 0.001	0.001^a^ ± 0.001	0.002^a^ ± 0.002

**Table 4:** Relative percentage of 18S OTUs Taxonomic composition at phylum level. Detected in the tests performed with the Ocean Sampling Day (OSD) standard procedure and with the autonomous Biosampler (IS-ABS) (mean ± standard deviation, n = 3). For IS-ABS two filtration pressures were selected (1 and 1.3 bars). Different superscript letters indicate significant (ANOVA, *P* < 0.05) differences among the three filtration procedures for each phylum.

	OSD	IS-ABS	
	≈1bar	1bar	1.3 bar
*Alveolata*	36^a^ ± 2	37^a^ ± 3	36^a^ ± 2
*Stramenopiles*	28^a^ ± 1	25^b^ ± 2	24^ab^ ± 4
*Archaeplastida*	18^a^ ± 1	18^a^ ± 4	22^a^ ± 6
*Opisthokonta*	10^a^ ± 3	12^a^ ± 6	11^a^ ± 3
*Hacrobia*	3.6^a^ ± 0.4	3.2^a^ ± 0.2	3^a^ ± 1
*Rhizaria*	2^a^ ± 1	4^a^ ± 2	1.7^a^ ± 0.3
*Apusozoa*	0.8^a^ ± 0.5	0.8^a^ ± 0.3	0.6^a^ ± 0.3
*Eukaryota unclassified*	0.5^a^ ± 0.4	0.5^a^ ± 0.4	0.6^a^ ± 0.5
*Amoebozoa*	0.4^a^ ± 0.3	0.6^a^ ± 0.2	0.5^a^ ± 0.1
*Excavata*	0.1^a^ ± 0.1	0.2^ab^ ± 0.1	0.3^b^ ± 0.1

Results showed that prokaryotic and eukaryotic taxonomic composition at higher taxonomic levels was not affected by the filtration pressures (1 bar *vs* 1.3 bar) applied in the IS-ABS.

#### 3.2.5 Performance at community lower taxonomy level

**Fig 11:**
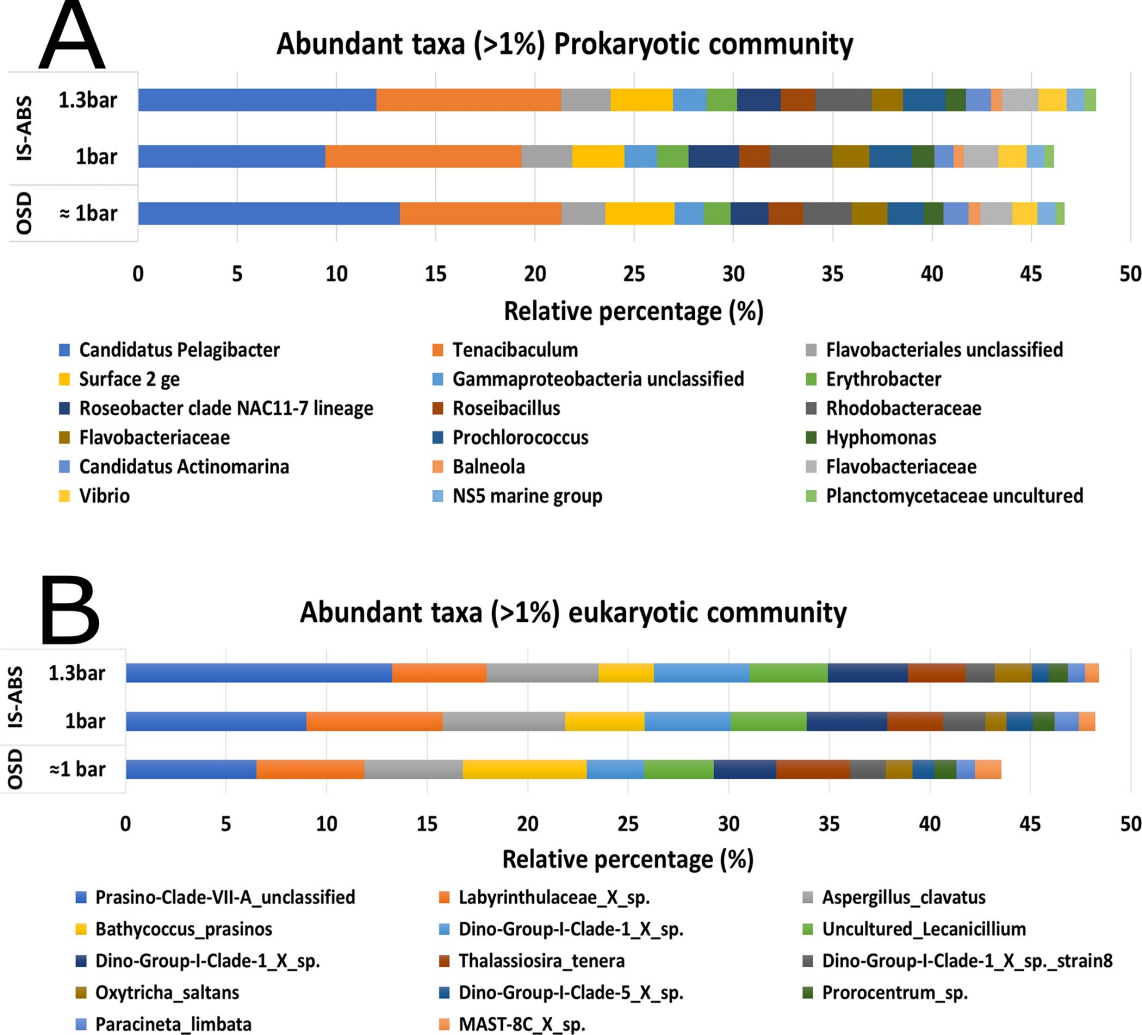
**Distribution of the abundant taxa (>1%) retrieved from the 16S rDNA (A) and 18S rDNA (B) OTUs taxonomic composition at lower taxonomic level.** Detected in the testes performed with the Ocean Sampling Day (OSD) standard procedure and with the autonomous biosampler (IS-ABS) (mean ± standard deviation, n = 3). For IS-ABS two filtration pressures were selected (1 and 1.3 bar). No significant differences (ANOVA, *P* ≥ 0.05) were observed among the three filtration procedures for the relative percentage of each genus.

A lower triangular resemblance matrix using Bray Curtis similarity was performed to identify potential effects of the different filtration procedures (OSD and IS-ABS). Prokaryotic (16S rDNA) community structure (Part A in S5 Fig) at OTUs level showed higher dissimilarities among the replicates (A, B and C) of each filtration procedure than between the type of filtration itself (OSD vs IS-ABS). Nevertheless, no significant (ANOVA, *P* ≥ 0.05) differences in Bacteria and Archaea genera were observed between the two filtration procedures used (Fig 11A).

Exploring the protistan community at lower taxonomic level we identified, among the most abundant taxa (with a relative abundance higher than 1%), big cell size groups belonging to micro/mesoplankton: Bacillariophycae, Ciliophora and Dinophyceae such as *Prorocentrum* sp. (1% of abundance) (dark green in Fig 11B). The same abundance of 1% was found for smaller photosynthetic groups, e.g. the picoeukaryotes, MAST-8C_X_sp (pink/yellow in Fig 11B). In addition, all the identified genera of both prokaryotic and eukaryotic communities were present in all samples, independently of the filtration system used and pressures applied (Fig 11).

Results showed minor and non-significant (Simprof, *P* ≥ 0.05) shifts in prokaryotic and eukaryotic communities at lower taxonomic composition level between the two filtration procedures (IS-ABS and standard OSD).

#### 3.2.6 Performance at rare species level

The reproducibility of the filtration procedures on rare (< 1%) microbiome diversity was evaluated by comparing several diversity indices, including the number of observed OTUs, Chao1, Shannon, Berger Parker dominance, Simpson’s evenness, and also the Good coverage (S4 Table). General trends in the diversity indices showed no significant (ANOVA, *P* ≥ 0.05) differences between the two filtration procedures.

Also, the convergence observed in the rarefaction curves reflected that almost all rare (< 1%) OTUs accumulated at a roughly constant rate as the number of reads increases (S6 Fig) and without differences between the filtration procedures.

The analysis of rare prokaryotic (16S rDNA; Part A in S7 Fig) and eukaryotic (18S rDNA; Part B in S7 Fig) community structure at phylum level did not show major shifts between the two filtration procedures (OSD and IS-ABS prototype). The same was true at OTUs level (lower taxonomic level). The lower triangular resemblance matrix showed that rare (<1%) prokaryotic (16S rDNA) community (Part A in S8 Fig), and eukaryotic (18S rDNA) community (Part B in S8 Fig) did not show major differences between OSD and IS-ABS prototype filtration procedures.

## 4. Discussion

Environmental DNA has been proposed as a new bio-monitoring tool [40], although best practice protocols and cost effective techniques are still under development [41]. In this study, the IS-ABS, a field prototype designed to monitor plankton microbiomes, was developed by a multidisciplinary team of engineers and biologists. Nowadays, automated sampling devices capable of conducting eDNA sampling and molecular-biological sensing *in situ* are a promising approach for resolving high spatial and temporal water monitoring in different aquatic environments [12].

Our IS-ABS is a small size and compact biosampler able to capture real time different size class of microplankton (from bacteria to protists) for posterior molecular analysis. One of the main advantages of the developed IS-ABS is to minimize artifacts associated with sample handling, maximizing sterile conditions and enabling almost immediate preservation of biological samples. Furthermore, the integration of the IS-ABS in different water observation systems (such as AUVs or fixed platforms) will substantially increase the biological surveillance capabilities through large scale temporal studies of microbial communities diversity and functions.

The IS-ABS filtration efficiency was validated by comparison with conventional manual sample collection based on standard laboratory filtration protocols described in MicroB3 OSD Handbook [15]. We performed high-throughput sequencing (HTS) metabarcoding to capture *in situ* aquatic microbiomes, analyzing the reproducibility of eDNA recovery and microbial diversity (detected respectively by 16S rDNA and 18S rDNA barcodes) in samples collected by both filtration methodologies.

Validation results showed that IS-ABS efficiency was similar to traditional methods (from the sampling collection, to filtration and subsequent downstream analysis), highlighting IS-ABS applicability as an innovative tool in aquatic eDNA bio-monitoring surveys.

### 4.1 Filtration

Our IS-ABS performs *in situ* water collection and filtration through Sterivex-GP filters, widely recognized as standard device and an optimal capture strategy for characterizing microbial communities [42,43]. Moreover, IS-ABS is capable of preserving microbial biomass in up to 16 sample filters per deployment and in conditions compatible with subsequent megasequencing studies.

The filtration in multiple filters adds at the same time both redundancy and statistical significance to the collected data for the metagenomics and metatranscriptomics analyses. This ability will allow researchers to link the identity and activity of the microbiomes present in the water column with biological function at the exact time of sampling [44]. The IS-ABS cartridge boxes were made in high-density polyethylene (HDPE 1000), a material, that maintains its properties at extreme temperatures. Thus, the boxes are also convenient for storage of samples in cryogenic conditions, which is another suitable method to preserve samples until further molecular analysis [45]. This allows long transport times (such as the ones occurring in a typical oceanographic campaign). Once in the lab, the individual Sterivex filters can be removed from the cartridge box for DNA/RNA extraction and subsequent sequencing. Also, the new IS-ABS overcomes some limitations of the traditional sampling with Niskin bottles and shipboard filtration, which are sample storage and transportation to home laboratory, and need for dedicated clean facilities for eDNA filtration. IS-ABS will therefore reduce operational costs and risks of sample deterioration/contamination during the sample and filtration process. Moreover, IS-ABS protocol is less time consuming compared to standard manual filtration since filtration time using the IS-ABS is substantially reduced.

### 4.2 DNA yield

Most studies recommend the use of 0.2 μm pore size to capture free-living microbial planktonic microbes [46,47]. Since few planktonic marine microbes likely pass through 0.2 μm porosity [48], we assume that most of the free-living microplankton present in the water sampled in this study retained on the filters tested.

Results showed that in average, DNA yield from samples collected by either filtration methodology was similar. Reducing the time between sampling and filtering is critical, to ensure sample replicability [49], and to avoid the risk of eDNA degradation, for instance due to prolonged exposure under higher temperatures when sampling remote cold-water locations [50,51]. Field-filtration performed by the IS-ABS tackles these logistical constrains. Moreover, the lower filtration times required (due to its higher flow rates) by IS-ABS is advantageous for maximum eDNA recovery, compared to manual filtration performed in the laboratory.

### 4.3 Alpha and Beta diversity

Diversity indexes are an indicator used by many researchers to compare and further characterize differences between communities [52]. Thus, we use these indexes to validate the prokaryotic and eukaryotic microbiome diversity recovered from IS-ABS system. Alpha diversity rarefaction curves calculated by both methodologies were close to saturation, but did not reach the plateau. Such results suggest the need for a deeper sampling effort to cover the whole prokaryotic and eukaryotic diversity. However, in terms of 16S and 18S community (OTU) richness, the number of OTUs detected were similar within the two filtration methods used. Chao1 index slightly variated between autonomous and manual filtrations and within the two filtration pressures (1 and 1.3 bar) tested for IS-ABS. This could be explained by the greater weight that Chao richness estimator gives to the low abundance species, as only the singletons and doubletons are used to estimate the number of missing species [53]. On the other hand, Shannon’s diversity as well as Berger Parker dominance and Simpson’s evenness indices showed in general no differences between the OSD and IS-ABS automatic filtration procedures. These results suggest that the latter indices are more influenced by dominance/abundance of OTUs (e.g. Shannon diversity index depends more on highly abundant OTUs than on species richness estimates), being more stable than the richness estimators and more reliable for comparison across various studies [54].

With regard to β-diversity, dendrograms generated from hierarchical analysis based on Bray–Curtis similarities showed that samples clustered by replicates rather than by filtration process. In fact, results showed higher dissimilarities between the replicates (A, B and C) rather than between the procedures of filtration itself (OSD vs IS-ABS). Differences between replicates, although not statistically significant, were observed for the dominant and rare microplankton community that could have been caused by inherent water mass biological heterogeneity [55].

### 4.4 Taxonomic composition

According to our results, the two filtration systems were equivalent, in terms of community compositions at higher as well as at lower taxonomic level of 16S rDNA and 18S rDNA, indicating that the procedures converged in equivalent results. The major taxa were evenly distributed in both datasets, reporting very similar proportional abundances.

Concerning the lowest taxonomic level of eukaryotic microplankton community (revealed via 18S rDNA analysis), the IS-ABS system did not change the relative abundance of genera, and samples from both filtration methods harbor both large (micro/mesoplankton) and small (picoplankton/nanoplankton) microorganisms. Unicellular eukaryotes, explored by 18S rDNA dataset, include a wide range of microorganisms, included diatoms, with fortified cell walls (e.g. *Thalassiosira* genus), as well as dinoflagellates with cellulosic thecal or thin-walled phytoplankton. Because, protists vary in size and firmness of cell wall, can be highly susceptible to cell breakage or loss during filtration process [56]. Results from this study showed no significant differences between filtration procedures (OSD and IS-ABS) at similar working pressures (≈1 bar) in the relative abundance of taxa with fragile membranes, like the case of *Micromonas* Clade-ABC, nor in phytoplankton taxa like *Thalassiosira tenera* (small but heavily silicified species possessing a linear areolar array). However, a tendency to lower *Thalassiosira tenera* relative abundance was observed in the IS-ABS, at a higher working pressure (1.3 bar) when compared with filtrations with IS-ABS at 1 bar, which might suggest that a relative small increase in filtration pressure can damage the heavy silica cell walls of unicellular eukaryotes.

Bacteria and archaea microbial cells targeted with 16S rDNA primers have a smaller size and are generally easier to lyse than individual microeukaryotes cells [57]. Moreover, prokaryotic diversity is dominated by a highly low abundance biosphere [58] representing a methodological challenge in terms of whole community recovery. Rare species (< 1% relative abundance) are increasingly recognized as crucial since they can have an over-proportional role in biogeochemical cycles and may be a hidden driver of microbiome function, such as in the response to organic pollutants [59,60]. Discovery of rare taxa and detection of previously unrecognized eukaryotic and prokaryotic microbiomes have been recently facilitated by HTS of DNA barcodes, e.g. on the Illumina MiSeq, Ion Torrent, or PacBio platforms [61,62]. Our results showed that, independently of the IS-ABS system filtration pressures applied, all genera of rare biosphere (< 1%), including prokaryotic and microeukaryotic communities, were present in all water samples. Thus, our findings showed that the IS-ABS is capable of detecting shifts in low relative abundant plankton groups.

Our IS-ABS tests at two different pressures (1 and 1.3 bar), showed highly similar results between eDNA recovered and microplankton prokaryotic and eukaryotic community structure. Although it would be important to deeply explore the impact of filtration pressures differences in eDNA retention, as well as on plankton microbial structure and rare biosphere. To our knowledge few studies are focused on this topic; hypothesizing that pressure is an influential factor that might lead to cell damage or breakage, with a consequent loss of DNA [63,64].

Results from the validation tests performed by the IS-ABS were very promising, indicating no relevant differences on the prokaryotic and protists diversity and composition analysis relative to standard filtration protocols. Therefore, our research describing the innovative IS-ABS workflow, for eDNA sampling and processing would provide an accurate, fast and affordable bio-tool for monitoring microbial communities in aquatic ecosystems.

## 5. Final remarks and future prospects

The novel IS-ABS is an autonomous biosampler, with the ability to collect eDNA samples for later genomic analysis. This study demonstrated a similar performance between the IS-ABS system and the standard manual protocol (OSD protocol) with respect to eDNA recovery and plankton microbiome diversity at prokaryotic and eukaryotic levels.

The IS-ABS is a small and compact system, making it very convenient to transport. Also, the IS-ABS is very easy and simple to use and integrates a user-friendly application to program sampling definitions. The major advantage of the IS-ABS is allowing autonomous *in situ* filtration and sample preservation of plankton microbial communities. Generally existing devices can only sample in the ocean (e.g. onboard an oceanographic research vessel), since they are too large, complex and expensive for widespread use. The IS-ABS represents a new resource for researchers interested in more accurate plankton microbial sampling; specially designed to be used, not only in oceanic research, but also in coastal, estuarine, riverine, lakes or aquaculture environments. Thus, our IS-ABS system can be successfully employed to increase spatial and temporal resolution of aquatic microbiome monitoring. It will represent a key complement to fixed and mobile (e.g. AUV) aquatic observation systems to tackle the biological knowledge gap in the understudied remote aquatic ecosystems.

## Supporting information

S1 Fig Examples of biosampler configuration and monitoring web pages. Two screenshots taken of the configuration web page. Top) Part of a configuration example of a water filtration mission. Bottom) Easy to read resume example of the next mission to be executed.
(DOCX)

S2 Fig Laboratorial OSD filtration apparatus. (a) Diaphragm vacuum pump. (b) Water waste collection bottle. (c) PowerVac Manifold. (d) Sterivex filters. (e) 50 mL sterile syringes.
(DOCX)

S3 Fig Mean rarefaction curves Calculated in the samples using either the Ocean Sampling Day filtration standard procedure (OSD) or the autonomous biosampler (IS-ABS) (mean ± standard deviation, n = 3). For IS-ABS two filtration pressures (1 and 1.3 bar) were used. These curves indicate the number of Operational taxonomic units (OTUs) observed (a) in the total 16S rDNA dataset amplicons for investigating the alpha diversity of prokaryotic communities; (b) in the total 18 rDNA dataset amplicons of eukaryotic communities. Error bars represent standard deviation.
(DOCX)

S4 Fig **Heatmap of the 16S rDNA (A) and 18S rDNA (B) Operational taxonomic units (OTUs) at phylum level.** Generated from relative abundance matrix obtained from the 16S rDNA Prokaryotic and the 18S rDNA Eukaryotic communities, respectively, in samples recovered using either the Ocean Sampling Day filtration standard procedure (OSD) or the autonomous biosampler (IS-ABS) (n = 3), at the same working pressure of 1.0 bar.
(DOCX)

S5 Fig **Dendogram from the 16S rDNA (A) and 18S rDNA (B) at the Operational taxonomic units (OTUs) level.** Dendogram generated from hierarchical analysis based on Bray–Curtis similarities of the lower triangular resemblance matrix obtained and using the Simprof test to verify significant differences (black) between clusters generated. Samples recovered using either the Ocean Sampling Day filtration standard procedure (OSD) or the autonomous biosampler (IS-ABS) (n = 3). For IS-ABS two filtration pressures were selected (1 and 1.3 bar).
(DOCX)

S6 Fig Mean rarefaction curves of rare OTUs. Calculated in the samples recovered using either the Ocean Sampling Day filtration standard procedure (OSD) or the autonomous biosampler (IS-ABS) (mean ± standard deviation, n = 3). For IS-ABS two filtration pressures (1 and 1.3 bar) were used. These curves indicate the number of Operational taxonomic units (OTUs) observed in the rare filtrated biosphere (<1% of the total 16S rDNA and 18S rDNA amplicons datasets) for investigating the alpha diversity of prokaryotic communities (a) and eukaryotic communities (b). Error bars represent standard deviation.
(DOCX)

S7 Fig **Heatmap of the rare (< 1%) 16S rDNA (A) and 18S rDNA (B) Operational taxonomic units (OTUs) at phylum level.** Generated from relative abundance matrix obtained from the rare 16S rDNA Prokaryotic (A) and 18S rDNA Eukaryotic (B) communities in samples recovered using either the Ocean Sampling Day filtration standard procedure (OSD) or the autonomous biosampler (IS-ABS) (n = 3), at the same working pressure of 1.0 bar.
(DOCX)

S8 Fig **Dendogram from the rare (< 1%) 16S rDNA (A) and 18S rDNA (B) at the Operational taxonomic units (OTUs) level.** Generated from hierarchical analysis based on Bray–Curtis similarities of the lower triangular resemblance matrix obtained and using the Simprof test to verify significant differences (black and full lines) between clusters generated. Samples recovered using either the Ocean Sampling Day filtration standard procedure (OSD) or the autonomous biosampler (IS-ABS) (n = 3). For IS-ABS two filtration pressures (1 and 1.3 bar) were selected.
(DOCX)

S1 Table Filtration time and average flow. Water filtered, for a total filtration volume of 2 L of sample and measured in fractions of 100 mL with the *in situ* autonomous biosampler (IS-ABS) at 0.8, 1.0, and 1.3 bar (average ± standard deviation, n = 3).
(DOCX)

S2 Table Overview of the 16S and 18S datasets. Datasets were generated from OSD standard methodologies and IS-ABS at 1 bar filtration pressure; and with IS-ABS at two different filtration pressures (1 and 1.3 bar). Different superscript letters indicate significant (ANOVA, *P* < 0.05) differences among the three filtration procedures in each parameter.
(DOCX)

S3 Table The number the rare (<1%) OTUs (97%) in the 16S and 18S rDNA. Detected across the different procedures (Ocean Sampling day (OSD) and *in situ* autonomous filtration prototype (IS-ABS); and different filtration pressures (1 and 1.3 bar). Information for each treatment replicates (A, B and C) and for total samples. Raw read pairs directly obtained from Illumina MiSeq sequencing platform, the sequence count after cleaning by mothur analysis pipeline, for each group. The different superscript letters show significant (ANOVA, *P* < 0.05) differences among filtration procedures.
(DOCX)

S4 Table Diversity indices for rare (<1%) 16S and 18S rDNA. Detected in the tests performed with the Ocean Sampling Day (OSD) standard procedure and with the autonomous DNA sampler (IS-ABS) (mean ± standard deviation, n = 3). For IS-ABS two filtration pressures were selected (1 and 1.3 bar). Different superscript letters indicate significant (ANOVA, *P* < 0.05) differences among the three filtration procedures for each diversity index.
(DOCX)
