# Supplementary material for: Development of an autonomous biosampler to capture in situ aquatic microbiomes
Source: PLoS One. 2019 May 15;14(5):e0216882. doi: 10.1371/journal.pone.0216882 (PMC6519839; doi:10.1371/journal.pone.0216882)

**Development of an autonomous biosampler to capture *in situ* aquatic microbiomes**

**S1 Fig. Examples of biosampler configuration and monitoring web pages.** Two screenshots taken of the configuration web page. Top) Part of a configuration example of a water filtration mission. Bottom) Easy to read resume example of the next mission to be executed.


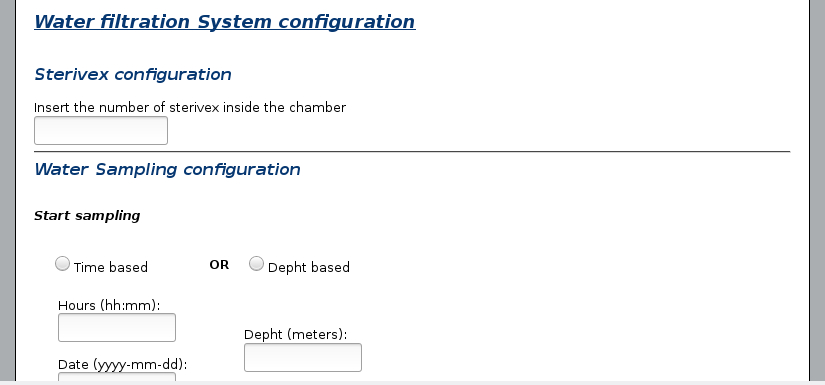


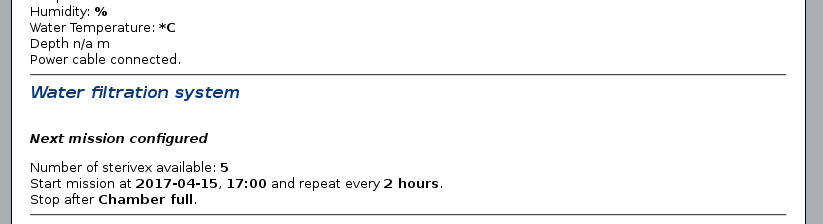

Supplement: S1 Fig — Two screenshots taken of the configuration web page. Top) Part of a configuration example of a water filtration mission. Bottom) Easy to read resume example of the next mission to be executed. (DOCX) [file pone.0216882.s001.docx]
