# Supplementary material for: Development of an autonomous biosampler to capture in situ aquatic microbiomes
Source: PLoS One. 2019 May 15;14(5):e0216882. doi: 10.1371/journal.pone.0216882 (PMC6519839; doi:10.1371/journal.pone.0216882)
Supplement: S2 Fig — (a) Diaphragm vacuum pump. (b) Water waste collection bottle. (c) PowerVac Manifold. (d) Sterivex filters. (e) 50 mL sterile syringes. (DOCX) [file pone.0216882.s002.docx]

**Development of an autonomous biosampler to capture *in situ* aquatic microbiomes**

**S2 Fig. Laboratorial OSD filtration apparatus.** (a) Diaphragm vacuum pump. (b) Water waste collection bottle. (c) PowerVac™ Manifold. (d) Sterivex filters. (e) 50 mL sterile syringes.
