# Supplementary material for: Development of an autonomous biosampler to capture in situ aquatic microbiomes
Source: PLoS One. 2019 May 15;14(5):e0216882. doi: 10.1371/journal.pone.0216882 (PMC6519839; doi:10.1371/journal.pone.0216882)

**Development of an autonomous biosampler to capture *in situ* aquatic microbiomes**

**S3 Fig. Mean rarefaction curves** Calculated in the samples using either the Ocean Sampling Day filtration standard procedure (OSD) or the autonomous biosampler (IS-ABS) (mean ± standard deviation, n = 3). For IS-ABS two filtration pressures (1 and 1.3 bar) were used. These curves indicate the number of Operational taxonomic units (OTUs) observed (a) in the total 16S rDNA dataset amplicons for investigating the alpha diversity of prokaryotic communities; (b) in the total 18 rDNA dataset amplicons of eukaryotic communities. Error bars represent standard deviation.


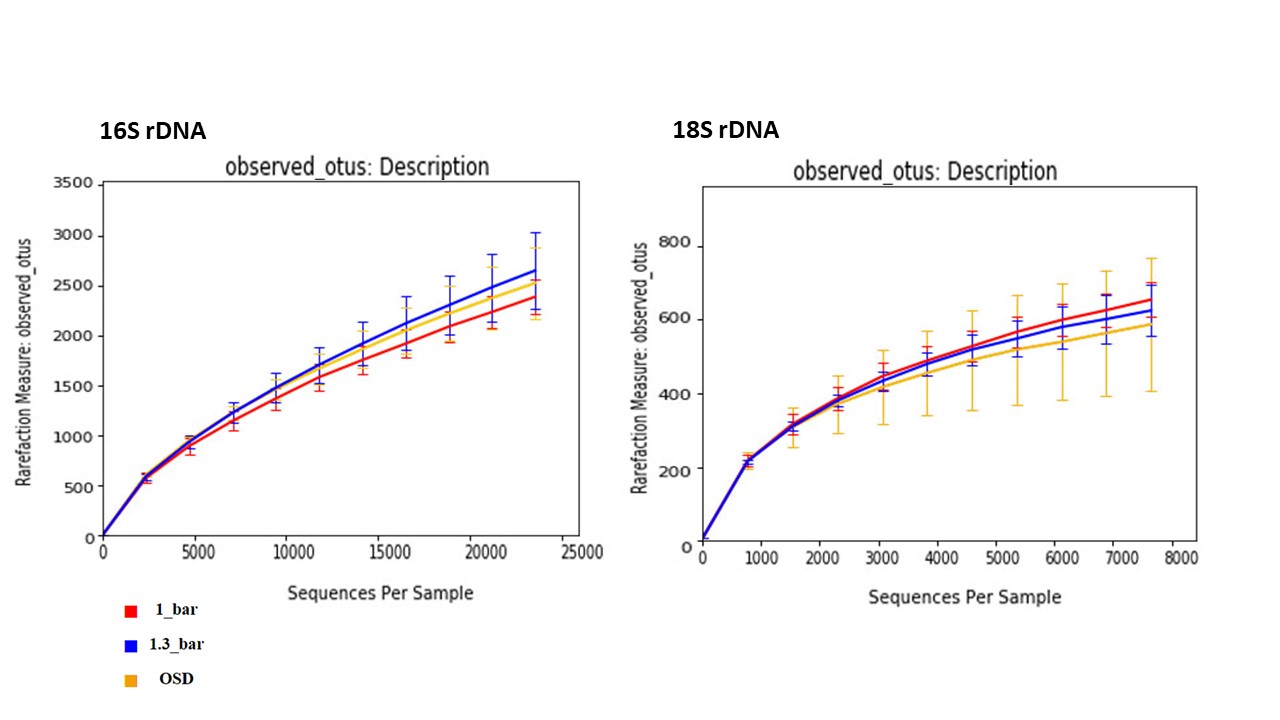

Supplement: S3 Fig — For IS-ABS two filtration pressures (1 and 1.3 bar) were used. These curves indicate the number of Operational taxonomic units (OTUs) observed (a) in the total 16S rDNA dataset amplicons for investigating the alpha diversity of prokaryotic communities; (b) in the total 18 rDNA dataset amplicons of eukaryotic communities. Error bars represent standard deviation. (DOCX) [file pone.0216882.s003.docx]
