# Supplementary material for: Development of an autonomous biosampler to capture in situ aquatic microbiomes
Source: PLoS One. 2019 May 15;14(5):e0216882. doi: 10.1371/journal.pone.0216882 (PMC6519839; doi:10.1371/journal.pone.0216882)

**Development of an autonomous biosampler to capture *in situ* aquatic microbiomes**

**S4 Fig. Heatmap of the 16S rDNA (A) and 18S rDNA (B) Operational taxonomic units (OTUs) at phylum level.** Generated from relative abundance matrix obtained from the 16S rDNA Prokaryotic and the 18S rDNA Eukaryotic communities, respectively, in samples recovered using either the Ocean Sampling Day filtration standard procedure (OSD) or the autonomous biosampler (IS-ABS) (n = 3), at the same working pressure of 1.0 bar.


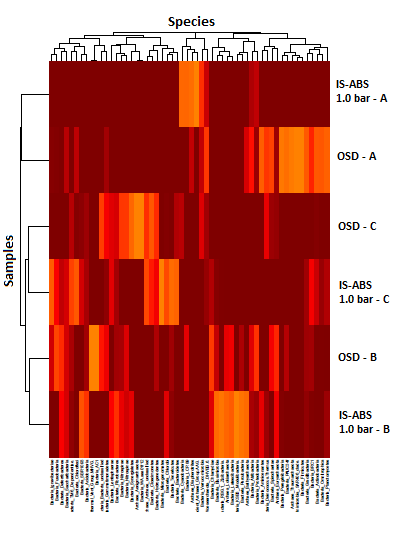


A

B


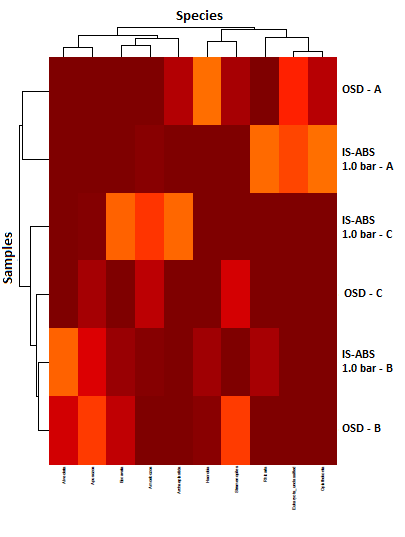

Supplement: S4 Fig — Heatmap of the 16S rDNA (A) and 18S rDNA (B) Operational taxonomic units (OTUs) at phylum level. Generated from relative abundance matrix obtained from the 16S rDNA Prokaryotic and the 18S rDNA Eukaryotic communities, respectively, in samples recovered using either the Ocean Sampling Day filtration standard procedure (OSD) or the autonomous biosampler (IS-ABS) (n = 3), at the same working pressure of 1.0 bar. (DOCX) [file pone.0216882.s004.docx]
