# Supplementary material for: Development of an autonomous biosampler to capture in situ aquatic microbiomes
Source: PLoS One. 2019 May 15;14(5):e0216882. doi: 10.1371/journal.pone.0216882 (PMC6519839; doi:10.1371/journal.pone.0216882)
Supplement: S6 Fig — Calculated in the samples recovered using either the Ocean Sampling Day filtration standard procedure (OSD) or the autonomous biosampler (IS-ABS) (mean ± standard deviation, n = 3). For IS-ABS two filtration pressures (1 and 1.3 bar) were used. These curves indicate the number of Operational taxonomic units (OTUs) observed in the rare filtrated biosphere (<1% of the total 16S rDNA and 18S rDNA amplicons datasets) for investigating the alpha diversity of prokaryotic communities (a) and eukaryotic communities (b). Error bars represent standard deviation. (DOCX) [file pone.0216882.s006.docx]

**Development of an autonomous biosampler to capture *in situ* aquatic microbiomes**

**S6 Fig. Mean rarefaction curves of rare OTUs.** Calculated in the samples recovered using either the Ocean Sampling Day filtration standard procedure (OSD) or the autonomous biosampler (IS-ABS) (mean ± standard deviation, n = 3). For IS-ABS two filtration pressures (1 and 1.3 bar) were used. These curves indicate the number of Operational taxonomic units (OTUs) observed in the rare filtrated biosphere (<1% of the total 16S rDNA and 18S rDNA amplicons datasets) for investigating the alpha diversity of prokaryotic communities (a) and eukaryotic communities (b). Error bars represent standard deviation.

**
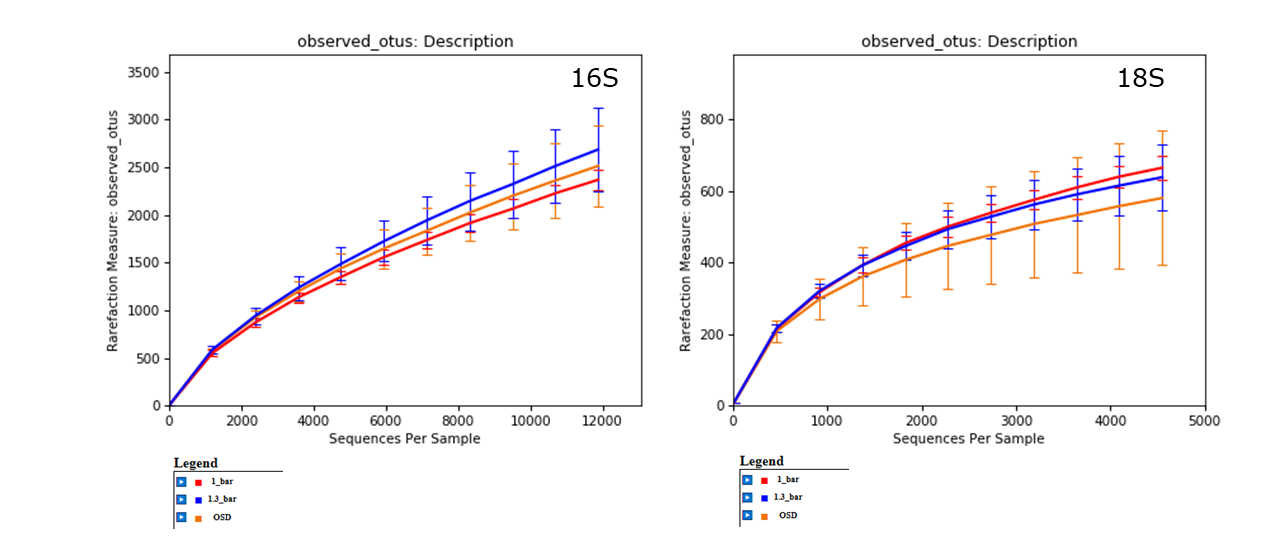
**
