# Supplementary material for: Development of an autonomous biosampler to capture in situ aquatic microbiomes
Source: PLoS One. 2019 May 15;14(5):e0216882. doi: 10.1371/journal.pone.0216882 (PMC6519839; doi:10.1371/journal.pone.0216882)
Supplement: S7 Fig — Heatmap of the rare (< 1%) 16S rDNA (A) and 18S rDNA (B) Operational taxonomic units (OTUs) at phylum level. Generated from relative abundance matrix obtained from the rare 16S rDNA Prokaryotic (A) and 18S rDNA Eukaryotic (B) communities in samples recovered using either the Ocean Sampling Day filtration standard procedure (OSD) or the autonomous biosampler (IS-ABS) (n = 3), at the same working pressure of 1.0 bar. (DOCX) [file pone.0216882.s007.docx]

**Development of an autonomous biosampler to capture *in situ* aquatic microbiomes**

**S7 Fig.** **Heatmap of the rare (< 1%) 16S rDNA (A) and 18S rDNA (B) Operational taxonomic units (OTUs) at phylum level.** Generated from relative abundance matrix obtained from the rare 16S rDNA Prokaryotic (A) and 18S rDNA Eukaryotic (B) communities in samples recovered using either the Ocean Sampling Day filtration standard procedure (OSD) or the autonomous biosampler (IS-ABS) (n = 3), at the same working pressure of 1.0 bar.

A


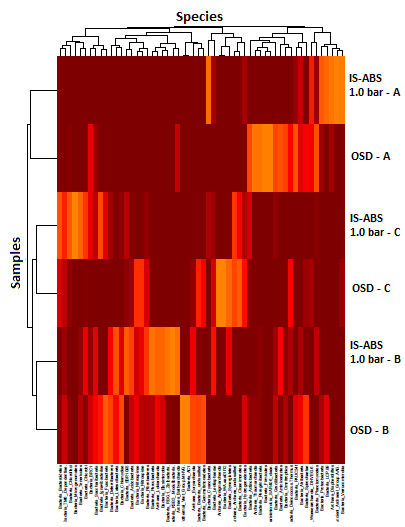


**
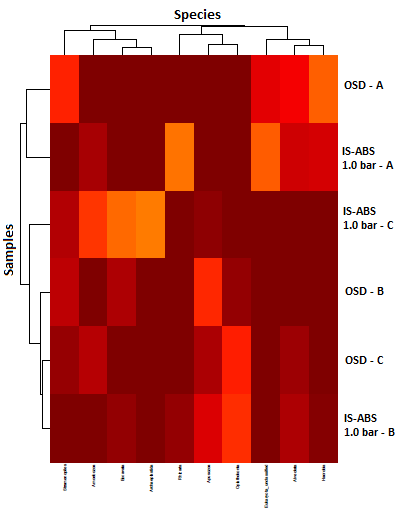
**

B
