# Supplementary material for: Development of an autonomous biosampler to capture in situ aquatic microbiomes
Source: PLoS One. 2019 May 15;14(5):e0216882. doi: 10.1371/journal.pone.0216882 (PMC6519839; doi:10.1371/journal.pone.0216882)
Supplement: S8 Fig — Dendogram from the rare (< 1%) 16S rDNA (A) and 18S rDNA (B) at the Operational taxonomic units (OTUs) level. Generated from hierarchical analysis based on Bray–Curtis similarities of the lower triangular resemblance matrix obtained and using the Simprof test to verify significant differences (black and full lines) between clusters generated. Samples recovered using either the Ocean Sampling Day filtration standard procedure (OSD) or the autonomous biosampler (IS-ABS) (n = 3). For IS-ABS two filtration pressures (1 and 1.3 bar) were selected. (DOCX) [file pone.0216882.s008.docx]

**Development of an autonomous biosampler to capture *in situ* aquatic microbiomes**

**S8 Fig.** **Dendogram from the rare (< 1%) 16S rDNA (A) and 18S rDNA (B) at the Operational taxonomic units (OTUs) level.** Generated from hierarchical analysis based on Bray–Curtis similarities of the lower triangular resemblance matrix obtained and using the Simprof test to verify significant differences (black and full lines) between clusters generated. Samples recovered using either the Ocean Sampling Day filtration standard procedure (OSD) or the autonomous biosampler (IS-ABS) (n = 3). For IS-ABS two filtration pressures (1 and 1.3 bar) were selected.


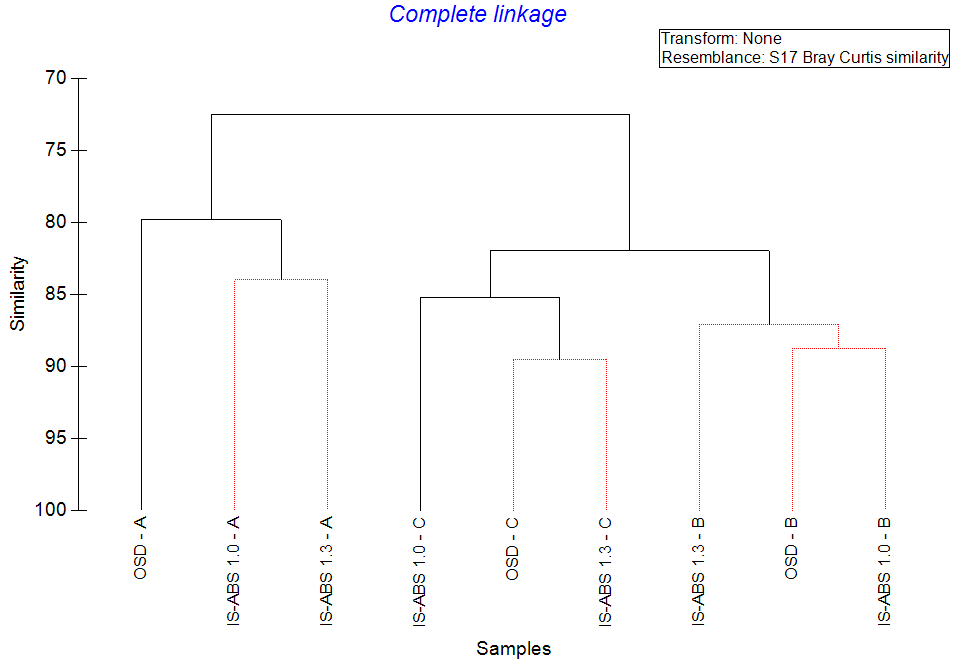


A


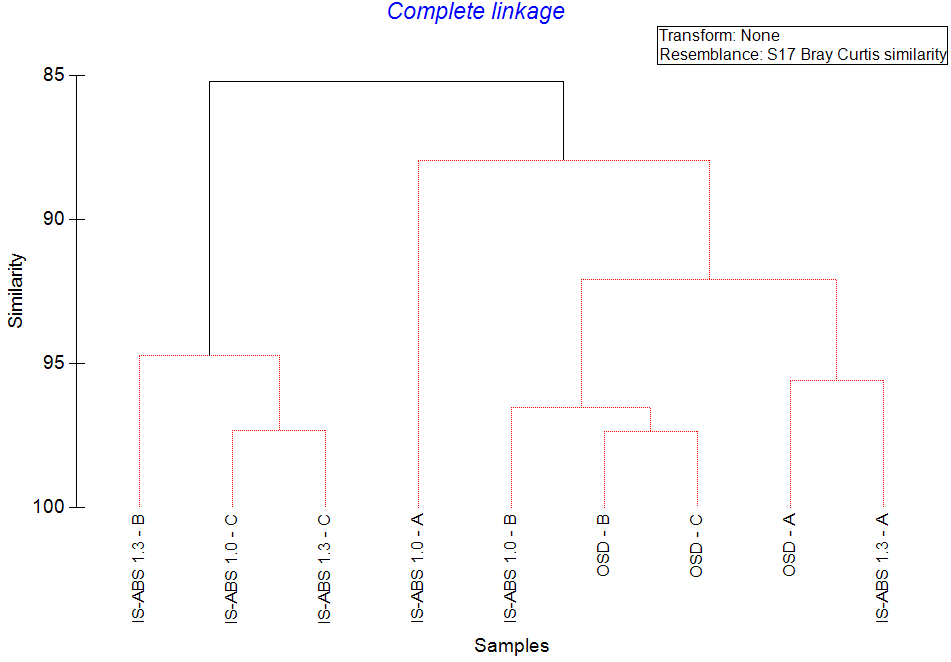


B
