# Supplementary material for: Development of an autonomous biosampler to capture in situ aquatic microbiomes
Source: PLoS One. 2019 May 15;14(5):e0216882. doi: 10.1371/journal.pone.0216882 (PMC6519839; doi:10.1371/journal.pone.0216882)
Supplement: S1 Table — Water filtered, for a total filtration volume of 2 L of sample and measured in fractions of 100 mL with the in situ autonomous biosampler (IS-ABS) at 0.8, 1.0, and 1.3 bar (average ± standard deviation, n = 3). (DOCX) [file pone.0216882.s009.docx]

**Development of an autonomous biosampler to capture *in situ* aquatic microbiomes**

**S1 Table**. **Filtration time and average flow**. Water filtered, for a total filtration volume of 2 L of sample and measured in fractions of 100 mL with the *in situ* autonomous biosampler (IS-ABS) at 0.8, 1.0, and 1.3 bars (average ± standard deviation, n = 3).

| **Volume intervals**  (mL) |  | **Pressure: 0.8 bar** | |  | **Pressure: 1.3 bar** | |  | **Pressure: 1.8 bar** | |
| --- | --- | --- | --- | --- | --- | --- | --- | --- | --- |
|  |  | **Time of filtration**  (seconds) | **Average Flow**  (mL min^-1^) |  | **Time of filtration**  (seconds) | **Average Flow**  (mL min^-1^) |  | **Time of filtration**  (seconds) | **Average Flow**  (mL min^-1^) |
| **0 - 100** |  | 73 ± 1 | 82 ± 2 |  | 55 ± 1 | 110 ± 2 |  | 42 ± 2 | 144 ± 5 |
| **100 - 200** |  | 77 ± 1 | 77 ± 1 |  | 54 ± 1 | 112 ± 1 |  | 44 ± 2 | 138 ± 5 |
| **200 - 300** |  | 80 ± 2 | 75 ± 2 |  | 55 ± 1 | 109 ± 2 |  | 44 ± 1 | 137 ± 2 |
| **300 - 400** |  | 81 ± 2 | 74 ± 2 |  | 56 ± 1 | 107 ± 2 |  | 45 ± 2 | 133 ± 5 |
| **400 - 500** |  | 84 ± 1 | 71 ± 1 |  | 58 ± 1 | 104 ± 2 |  | 46 ± 2 | 131 ± 5 |
| **500 - 600** |  | 86 ± 2 | 70 ± 1 |  | 59 ± 1 | 101 ± 2 |  | 48 ± 3 | 124 ± 6 |
| **600 - 700** |  | 89 ± 0 | 68 ± 0 |  | 61 ± 1 | 99 ± 2 |  | 48 ± 3 | 126 ± 7 |
| **700 - 800** |  | 92 ± 0 | 65 ± 0 |  | 62 ± 1 | 97 ± 2 |  | 53 ± 2 | 114 ± 5 |
| **800 - 900** |  | 95 ± 1 | 63 ± 1 |  | 65 ± 2 | 93 ± 3 |  | 51 ± 1 | 118 ± 3 |
| **900 - 1000** |  | 98 ± 1 | 61 ± 1 |  | 67 ± 2 | 90 ± 3 |  | 54 ± 2 | 112 ± 3 |
| **1000 - 1100** |  | 101 ± 1 | 59 ± 1 |  | 69 ± 3 | 87 ± 4 |  | 56 ± 3 | 108 ± 6 |
| **1100 - 1200** |  | 105 ± 0 | 57 ± 0 |  | 71 ± 3 | 85 ± 3 |  | 57 ± 2 | 105 ± 4 |
| **1200 - 1300** |  | 112 ± 3 | 53 ± 1 |  | 73 ± 3 | 82 ± 4 |  | 59 ± 3 | 102 ± 6 |
| **1300 - 1400** |  | 117 ± 2 | 51 ± 1 |  | 76 ± 4 | 79 ± 4 |  | 63 ± 3 | 95 ± 4 |
| **1400 - 1500** |  | 122 ± 1 | 49 ± 1 |  | 79 ± 5 | 76 ± 5 |  | 66 ± 3 | 92 ± 5 |
| **1500 - 1600** |  | 128 ± 2 | 47 ± 1 |  | 84 ± 5 | 72 ± 5 |  | 69 ± 4 | 88 ± 5 |
| **1600 - 1700** |  | 135 ± 3 | 44 ± 1 |  | 88 ± 7 | 69 ± 5 |  | 72 ± 4 | 84 ± 5 |
| **1700 - 1800** |  | 147 ± 4 | 41 ± 1 |  | 91 ± 7 | 66 ± 5 |  | 76 ± 7 | 79 ± 7 |
| **1800 - 1900** |  | 156 ± 2 | 38 ± 0 |  | 96 ± 8 | 63 ± 5 |  | 82 ± 7 | 73 ± 7 |
| **1900 - 2000** |  | 168 ± 1 | 36 ± 0 |  | 102 ± 10 | 59 ± 6 |  | 87 ± 9 | 69 ± 7 |
