# Supplementary material for: Development of an autonomous biosampler to capture in situ aquatic microbiomes
Source: PLoS One. 2019 May 15;14(5):e0216882. doi: 10.1371/journal.pone.0216882 (PMC6519839; doi:10.1371/journal.pone.0216882)
Supplement: S2 Table — Datasets were generated from OSD standard methodologies and IS-ABS at 1 bar filtration pressure; and with IS-ABS at two different filtration pressures (1 and 1.3 bar). Different superscript letters indicate significant (ANOVA, P < 0.05) differences among the three filtration procedures in each parameter. (DOCX) [file pone.0216882.s010.docx]

**Development of an autonomous biosampler to capture *in situ* aquatic microbiomes**

**S2 Table.** **Overview of the 16S and 18S datasets**. Datasets were generated from OSD standard methodologies and IS-ABS at 1 bar filtration pressure; and with IS-ABS at two different filtration pressures (1 and 1.3 bar). Different superscript letters indicate significant (ANOVA, *P* < 0.05) differences among the three filtration procedures in each parameter.

|  |  | **OSD** | **IS-ABS** | |
| --- | --- | --- | --- | --- |
|  |  | **≈1bar** | **1bar** | **1.3 bar** |
| **16S rDNA** | Raw paired-end  Reads^#^ | 78107^a^ ± 22162 | 53549^a^ ± 8106 | 48570^a^ ± 18049 |
|  | Unique reads after filtering^§^ | 63424^a^ ± 28551 | 47567^a^ ± 7453 | 43328^a^ ± 16119 |
|  | OTUs clustered at 97%^£^ | 52369^a^ ± 19904 | 41085^a^ ± 5173 | 34889^a^ ± 10247 |
| **18S rDNA** | Raw paired-end  Reads^#^ | 30177^a^ ± 20852 | 22510^a^ ± 8476 | 36019^a^ ± 27587 |
|  | Unique reads after filtering^§^ | 25776^a^ ± 17626 | 19195^a^ ± 7206 | 30710^a^ ± 23700 |
|  | OTUs clustered at 97%^£^ | 18044^a^ ± 11812 | 13328^a^ ± 3921 | 18536^a^ ± 10160 |
|  | ^#^Total number of paired-end sequences | | | |
|  | ^§^Unique sequences left after quality control | | | |
|  | ^£^OTUs obtained at 97% clustering after Metazoa and singletons removal | | | |
