# Supplementary material for: Development of an autonomous biosampler to capture in situ aquatic microbiomes
Source: PLoS One. 2019 May 15;14(5):e0216882. doi: 10.1371/journal.pone.0216882 (PMC6519839; doi:10.1371/journal.pone.0216882)
Supplement: S3 Table — Detected across the different procedures (Ocean Sampling day (OSD) and in situ autonomous filtration prototype (IS-ABS); and different filtration pressures (1 and 1.3 bar). Information for each treatment replicates (A, B and C) and for total samples. Raw read pairs directly obtained from Illumina MiSeq sequencing platform, the sequence count after cleaning by mothur analysis pipeline, for each group. The different superscript letters show significant (ANOVA, P < 0.05) differences among filtration procedures. (DOCX) [file pone.0216882.s011.docx]

**Development of an autonomous biosampler to capture *in situ* aquatic microbiomes**

**S3 Table. Number of rare (<1%) OTUs (97%) in the 16S and 18S rDNA.** Rare OTUs detected in the tests performed with the Ocean Sampling Day (OSD) standard procedure and with the autonomous biosampler (IS-ABS) (mean ± standard deviation, n = 3). For IS-ABS two filtration pressures were selected (1 and 1.3 bar). OTUs obtained at 97% clustering after Metazoa and singletons removal. Different superscript letters indicate significant (ANOVA, *P* < 0.05) differences among the three filtration procedures.

|  |  | **OSD** | **IS-ABS** | |
| --- | --- | --- | --- | --- |
|  | **Pressure** | **≈1bar** | **1bar** | **1.3 bar** |
| 16S rDNA | OTUs clustered at 97% | 26500^a^ ± 7880 | 20594^a^ ± 1445 | 17068^a^ ± 4587 |
| 18S rDNA | OTUs clustered at 97% | 10425^a^ ± 6430 | 7246^a^ ± 2468 | 9939^a^ ± 5324 |
