# Supplementary material for: Development of an autonomous biosampler to capture in situ aquatic microbiomes
Source: PLoS One. 2019 May 15;14(5):e0216882. doi: 10.1371/journal.pone.0216882 (PMC6519839; doi:10.1371/journal.pone.0216882)
Supplement: S4 Table — Detected in the tests performed with the Ocean Sampling Day (OSD) standard procedure and with the autonomous DNA sampler (IS-ABS) (mean ± standard deviation, n = 3). For IS-ABS two filtration pressures were selected (1 and 1.3 bar). Different superscript letters indicate significant (ANOVA, P < 0.05) differences among the three filtration procedures for each diversity index. (DOCX) [file pone.0216882.s012.docx]

**Development of an autonomous biosampler to capture *in situ* aquatic microbiomes**

**S4 Table.  Diversity indices for rare (<1%) 16S and 18S rDNA**. Detected in the tests performed with the Ocean Sampling Day (OSD) standard procedure and with the autonomous DNA sampler (IS-ABS) (mean ± standard deviation, n = 3). For IS-ABS two filtration pressures were selected (1 and 1.3 bar). Different superscript letters indicate significant (ANOVA, *P* < 0.05) differences among the three filtration procedures for each diversity index.

|  | **Diversity indices** | **OSD** | **IS-ABS** | |
| --- | --- | --- | --- | --- |
|  |  | **≈1bar** | **1bar** | **1.3 bar** |
| **16S rDNA** | Observed OTUs | 2531^a^ ± 558 | 2375^a^ ± 132 | 2680^a^ ± 516 |
|  | Chao1 | 6338^a^ ± 2789 | 5494^a^ ± 637 | 7202^a^ ± 3218 |
|  | Shannon index | 9.5^a^ ± 0.2 | 9.3^a^ ± 0.3 | 9.5^a^ ± 0.3 |
|  | Berger Parker | 0.024^a^ ± 0.002 | 0.03^a^ ± 0.01 | 0.03^a^ ± 0.01 |
|  | Simpson’s evenness | 0.10^a^ ± 0.02 | 0.09^a^ ± 0.03 | 0.09^a^ ± 0.00 |
|  | Good coverage | 0.87^a^ ± 0.05 | 0.88^a^ ± 0.01 | 0.86^a^ ± 0.04 |
| **18S rDNA** | Observed OTUs | 585^a^ ± 236 | 674^a^ ± 49 | 639^a^ ± 106 |
|  | Chao1 | 828^a^ ± 430 | 944^a^ ± 80 | 841^a^ ± 209 |
|  | Shannon index | 7.9^a^ ± 0.5 | 8.0^a^ ± 0.3 | 8.0^a^ ± 0.2 |
|  | Berger Parker | 0.025^a^ ± 0.005 | 0.05^a^ ± 0.04 | 0.03^a^ ± 0.01 |
|  | Simpson’s evenness | 0.26^a^ ± 0.07 | 0.18^a^ ± 0.06 | 0.22^a^ ± 0.04 |
|  | Good coverage | 0.95^a^ ± 0.03 | 0.94^a^ ± 0.01 | 0.95^a^ ± 0.02 |
